# Does it matter how you ask? Self-reported emotions to depictions of need-of-help and social context

**DOI:** 10.1186/s40359-015-0066-3

**Published:** 2015-04-07

**Authors:** Aenne A Brielmann, Margarita Stolarova

**Affiliations:** Department of Psychology and Zukunftskolleg, University of Konstanz, Universitaetsstrasse 10, Konstanz, 78464 Germany

**Keywords:** Social perception, Pleasantness, Unpleasantness, Emotional experience, Self-assessment manikin (SAM), Assessment methodology, Helping, Need-of-help, Arousal, Valence

## Abstract

**Background:**

When humans observe other people’s emotions they not only can relate but also experience similar affective states. This capability is seen as a precondition for helping and other prosocial behaviors. Our study aims to quantify the influence of help-related picture content on subjectively experienced affect. It also assesses the impact of different scales on the way people rate their emotional state.

**Methods:**

The participants (*N*=242) of this study were shown stimuli with help-related content. In the first subset, half the drawings depicted a child or a bird needing help to reach a simple goal. The other drawings depicted situations where the goal was achieved. The second subset showed adults either actively helping a child or as passive bystanders. We created control conditions by including pictures of the adults on their own. Participants were asked to report their affective responses to the stimuli using two types of 9-point scales. For one half of the pictures, scales of arousal (calm to excited) and of bipolar valence (unhappy to happy) were employed; for the other half, unipolar scales of pleasantness and unpleasantness (strong to absent) were used.

**Results:**

Even non-dramatic depictions of simple need-of-help situations were rated systematically lower in valence, higher in arousal, less pleasant and more unpleasant than corresponding pictures with the child or bird not needing help. The presence of a child and adult together increased pleasantness ratings compared to pictures in which they were depicted alone. Arousal was lower for pictures showing only an adult than for those including a child. Depictions of active helping were rated similarly to pictures showing a passive adult bystander, when the need-of-help was resolved. Aggregated unipolar pleasantness and unpleasantness ratings accounted well for arousal and even better for bipolar valence ratings and for content effects on them.

**Conclusion:**

This is the first study to report upon the meaningful impact of harmless need-of-help content on self-reported emotional experience. It provides the basis for further investigating the links between subjective emotional experience and active prosocial behavior. It also builds upon recent findings on the correspondence between emotional ratings on bipolar and unipolar scales.

**Electronic supplementary material:**

The online version of this article (doi:10.1186/s40359-015-0066-3) contains supplementary material, which is available to authorized users.

## Background

Emotions are thought to play a key role at multiple levels of social cognition and behavior (Keltner and Haidt 1999). As noted by Gallese and colleagues “we do not just ‘see’ or ‘hear’ an action or an emotion. Side by side with the sensory description of the observed social stimuli, internal representations of the state associated with these actions or emotions are evoked in the observer, ‘as if’ they were performing a similar action or experiencing a similar emotion.” (Gallese et al. 2004). So far this general assumption has not been investigated for the specific case of simple, everyday need-of-help. Do people experience and report unpleasant emotions when seeing someone needing help to reach a simple goal, e.g. to open a door? And could this be a valid reason for prosocial behavior, such as helping?

Even though there is evidence that some emotional activation motivates helping (e.g. pointed out in Schroeder et al. 1995, chapter 3), research has so far been vague about how a person’s emotional experience changes when he or she sees somebody in need. Previous studies have told us relatively little about what people actually feel when they witness others in need because they have tended to examine the general disposition of humans for empathy (e.g. Carlo et al. 1993; Fabes et al. 1999; Prot et al. 2014), inferred further emotional involvement from brain activity (Masten et al. 2011; Rameson et al. 2012), or measured current empathic responses on scales encompassing a variety of adjectives or statements (e.g. Cao 1993; Fabes et al. 2006; Fischer et al. 2010; Sze et al. 2012). The problem with the latter approach is that it makes arbitrary assumptions about which emotions can be considered distinct. Therefore, this approach cannot make any precise claims about the basic affective processes underlying the perception of need-of-help (see Barret 2006 for discussion).

Not only empirical findings but also theoretical approaches fail to specify the exact nature of changes in emotional experience in response to the need of others. Several theories in social psychology assume that different aspects of emotion are affected when the distress of others is observed. For example, the arousal-cost-reward model proposes that arousal is heightened when people see someone in need-of-help (Dovidio 1991; Fischer et al. 2006; Piliavin et al. 1982; Schroeder et al. 1995). This model implies that arousal reflects an unpleasant experience. Therefore, “arousal” as used in the arousal-cost-reward model should not be confused or equated with the same term used by researchers in the domain of core affect (e.g. Bradley and Lang 1994), where arousal is an affect dimension orthogonal to the experience of bipolar pleasantness and unpleasantness, termed valence (Russell 1979, see Barrett et al. 2009 for a recent discussion). This interpretation of arousal as an aspect of core affect that is largely independent from valence and can accompany both pleasant and unpleasant emotions has dominated psychophysiological research on emotion perception in recent decades (see e.g. Anders et al. 2004; Bradley and Lang 1994, 2000; Keil et al. 2003; Wilson-Mendenhall et al. 2013). By contrast, the arousal-cost reward model refers to a concept which has also been termed “empathic arousal” by researchers studying prosocial behavior (Hoffman 1981) and is explicitly said to contain an unpleasant, aversive component (Batson and Shaw 1991). Similarly, the negative state relief theory claims that seeing others in need-of-help elicits unpleasant feelings (initially proposed by Cialdini et al. 1973). Hence, even though social perception and emotion perception research favor different interpretations of core affect with regard to the relation between arousal and valence in particular, there is some theoretical agreement regarding the unpleasant nature of emotions that is thought to be elicited by others’ need for help, a notion that has not yet been tested empirically.

At least three different combinable processes can be proposed to the link emotional responses and prosocial behavior. First, it could be that people are intrinsically motivated to decrease *others’* distress and thus also their own. According to this view, the act of helping per se does not necessarily influence observers’ (and potential helpers’) affect, but the relief of the need-of-help of others does (Batson and Shaw 1991). Secondly, prosocial behavior per se can be rewarding (Lee 2008; Schroeder et al. 1995). According to this view, an observer has to actually execute the act of helping, merely seeing someone being helped is not enough to affect an observer’s emotional state, at least not to the same degree. Thirdly, the act of helping could generally be valued positively over the removal of need. Some evidence for this view can be found in studies showing that infants prefer agents that act prosocially to those which act in an antisocial manner (e.g. Hamlin and Wynn 2011). According to this last explanation, just seeing an act of helping should elicit positive emotions. This response should be discernible from the difference between seeing someone in need and someone not in need. It has not yet been investigated whether the observation of prosocial acts elicits specific emotional responses. In fact, many study designs do not allow for the differentiation between these components, since the relief of the need is often confounded with the prosocial action (whether a person carries this out or observes it, as e.g. in Weller and Hansen Lagattuta 2013). In this study, specific picture categories were created to differentiate between the impact of need-of-help, relieved need-of-help, active helping behavior and variations of social context.

In sum, there is evidence that emotional experiences contribute to prosocial behavior. Yet, it remains unclear whether it is the need-of-help, its relief or the act of helping that triggers certain emotional responses. When need-of-help content has been used to induce empathic responses in previous research, the stimuli were mostly dramatic situations, such as war, violence, or serious accidents (e.g. Stocks et al. 2009; Sze et al. 2012). Similarly, the very early behavior research on the factors promoting or hindering active help involved mostly persons in urgent need-of-help, such as people screaming while being abducted (for a review see Fischer et al. 2011). These stimulus materials and experimental designs mostly covered a small range of potentially relevant need-of-help situations and did not take into account that everyday prosocial actions often relate to simple needs, e.g. needing help to open a door or reach a top shelf. Recent behavioral studies, especially those investigating the ontogeny of prosocial behavior in children, have focused more on such trivial problems, such as not being able to reach a fallen object (e.g. Svetlova et al. 2010; Warneken and Tomasello 2007) and have thus expanded research to include everyday action. One specific aim of the present study is to assess how emotional experience is affected by seeing others in need-of-help and by seeing an act of helping particularly in trivial situations, in contrast to dramatic or dangerous ones. We have sought to clarify what changes in affect bear the potential to foster everyday prosocial behavior, rather than heroic action. The stimuli we used show situations that often lead to helping behavior in real life; they are simple depictions of people reaching or not reaching a goal, without any danger or substantial discomfort. By intentionally omitting pictures that were extreme in valence or arousal, we expected participants would create a frame of reference that reflected their subjective experience of low intensity visual stimuli. We assumed that meaningful effects of content found for a set of neutral, low arousing and perceptually similar stimuli would provide evidence that even trivial need-of-help situations elicit systematic affective responses, a previously uninvestigated hypothesis. We sought to create visual stimuli that do not elicit high arousal by controlling both their perceptual features (black-and-white drawings rather than realistic photographs) and content (trivial everyday situations, not dramatic events). Pictures showing birds added variation to the stimulus set and partially allowed us to assess whether emotional responses accompanying need-of-help perception are restricted to humans in species-specific situations.

In order to quantify core affect responses linked to help-related content, participants were asked to rate their subjective emotional response to a picture according to two dimensions. We used the Self-Assessment Manikin-scales (SAMs, Lang 1980), which are a popular tool for collecting such subjective emotional ratings. Employing these rating scales allowed us to relate the present findings to a vast body of literature on perception of emotional stimuli (e.g. Bradley and Lang 1994; Grühn and Scheibe 2008; McManis et al.^2001^). This conventional approach relies on the assumption that subjective emotional experiences can be unambiguously decomposed into arousal and bipolar valence. This idea has found support in numerous psychophysiological studies (e.g. Bradley and Lang 1994; Keil et al. 2003, for an extensive review see Bradley and Lang 2000), and also neuroimaging studies (Anders et al. 2004; Wilson-Mendenhall et al. 2013). However, as Barrett and Bliss-Moreau (Barrett et al. 2009) have pointed out: “[…] the structure of felt experience will not correspond to the brain processes that produced those experiences in a one-to-one fashion.” A recent study suggests that self-reported emotions are reflected in the intensity of pleasant and unpleasant feelings (Kron et al. 2013). In it mean differences between pleasantness and unpleasantness ratings of IAPS (International Affective Picture System) pictures are highly correlated with mean valence ratings. It suggests that mean arousal ratings can largely be explained by the sum of pleasantness and unpleasantness ratings. Furthermore, many studies using unipolar rating scales of pleasantness and unpleasantness have demonstrated that participants report mixed feelings, especially with regard to stimuli termed as relatively neutral, when valence and arousal ratings are considered (Hemenover and Schimmack 2007; Kreibig et al. 2013; Kron et al. 2013; Larsen and Green 2013; Schimmack et al. 2001; Schimmack and Colcombe 2007), which is an aspect that is not accessible when valence and arousal scales are used. Taking as a basis this emerging debate on the usefulness of scale type for measuring self-reported emotions, especially with regard to relatively neutral pictures, we used both types of rating scales - arousal and bipolar valence and pleasantness and unpleasantness - in a within-subject design. We attempted thus to expand on the methodological findings obtained with the IAPS (Ito et al. 1998; Kron et al. 2013) and develop current knowledge to support the assumption that self-reported arousal and bipolar valence can be understood as aggregated intensity of pleasant and unpleasant feelings with regard to relatively neutral and harmless depictions. An additional goal of the present study was to compare the content related effects obtained with both scales, in order to determine to what extend the two scale types can be used interchangeably.

Our study has two main goals. 1) Our content-related research question asks how self-reported emotional experiences relate to the perception of help-related pictures and variations of social context, including depictions of active helping. 2) Methodologically, we aim to expand research on the differences and commonalities of unipolar pleasantness and unpleasantness compared to arousal and bipolar valence rating scales using stimuli that could be expected to elicit only moderate emotional responses. Moreover, we are able to compare content-related results measured on arousal and bipolar valence scales to ones obtained with unipolar pleasantness and unpleasantness scales. Thereby, we provide an extended replication of recent findings reported by Kron and colleagues (Kron et al. 2013).

## Methods

### Ethics approval

This study was officially approved by the Ethics Committee of the University of Konstanz on 31st July 2013. It was also approved by the Dean of the Faculty of Society and Economics, Rhine-Waal University of Applied Science (1*st* October 2013). All participants signed written informed consent according to the Declaration of Helsinki.

### Stimuli

We used an extension of the NeoHelp stimulus set developed by the authors (Brielmann and Stolarova 2014) to investigate the perception of help-related content. All pictures were black-and-white drawings of 800 × 800 px in size showing variations of 10 different everyday situations (see Additional file 1).

**Figure 1:**
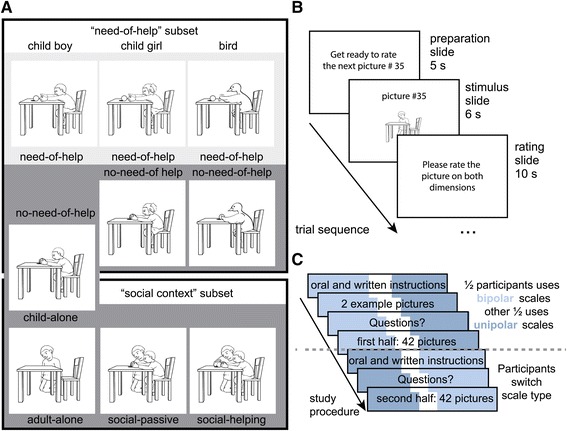
Example stimuli, trial and study sequence. Stimuli for each of ten situations were created in two subsets **(A)**: the “need-of-help” subset (top) consisted of need-of-help / no-need-of-help picture pairs showing a boy/toddler, a girl, or a bird each in the same situation. The “social context” subset (bottom) included 4 picture categories with varying contexts. Need-of-help pictures are shown on light gray, those showing no-need-of-help on darker gray backgrounds. Note that ratings of one picture per situation were analyzed as part of both subsets, i.e. the child boy “no-need-of-help”/“child-alone” picture. Trial sequence was pseudo randomized and was the same for all 84 pictures (**B**, see Additional file 1). The study was divided in two halves **(C)**.

The complete stimulus set was comprised of two parts, each focusing on one aspect of helping. The first picture subset, which we will refer to as “need-of-help” subset, was designed to investigate the perception of need-of-help in everyday non-dramatic situations. All pictures were created in pairs; one of them showed an agent needing help in a harmless everyday situation (e.g. trying to open a door), its counterpart showed the same agent achieving the same goal. In order to investigate whether emotional responses to need-of-help depictions are human-specific and restricted to realistic situations, there were at least two different need-of-help/no-need-of-help picture pairs for each situation; one of a child and one of a bird. For all situations showing a child with an identifiable gender (i.e. no toddlers) two pairs of child-pictures varying in gender were included. In sum, there were boy and girl picture pairs for seven situations, picture pairs of toddlers for three situations, and a corresponding picture pair showing birds for all ten situations. The “need-of-help” picture subset consisted of 54 pictures (see Figure 1 for illustration and an example situation).

The second - “social context” - picture subset was created to assess the self-reported emotional experience of active helping behavior. Control pictures were included to differentiate between the effects of active helping and those of relieved need and social context. It included four picture variations for each of the ten different situations (see Additional file 1) derived from one another to fit the following four categories: “child-alone”, “adult-alone”, social passive and social helping. The first category consisted of 10 pictures from the “need-of-help” subset, which showed a boy or toddler who reaches a goal and thus does not need help (in the context of this subset referred to as “child alone”). The “adult-alone” category consisted of 10 pictures showing an adult as well as other objects (e.g. a table, door, etc.). The 10 pictures comprising the “social passive” category were based on the “child-alone” pictures but also included the same adult as in the “adult-alone” picture. Finally, 10 pictures showing an adult actively helping a child to reach a goal made up the “social-helping” category. The “social context” subset consisted of 40 pictures in total. The only picture category belonging to both picture subsets was the one showing a boy or toddler alone and not needing help to reach a goal.

In sum, participants were shown a total of 84 different pictures, 44 of which belonged exclusively to the “need-of-help” subset, 30 of which belonged exclusively to the “social context” subset and 10 pictures, which belonged to both subsets. An overview of all pictures for one example situation is given in Figure 1 A. The complete set of stimuli is available as additional.xls file (see Additional file 1).

### Rating material

Paper-and-pencil rating booklets were provided to all participants. They all included written instructions and were divided into three parts, with changes of paper color indicating the change in rating scales’ type. To begin with, the participants’ gender attributions in response to the 30 pictures showing adults were assessed for a separate study and the results are not part of this present report. Then, self-reported emotional ratings were collected. Participants used scales comprising the SAM symbols and provided two ratings per picture. For half of the stimuli, participants used the traditional bipolar SAM scales with the two dimensions: bipolar valence (unhappy to happy) and arousal (calm to excited). For the other half, they used two unipolar scales for the dimensions pleasant and unpleasant feelings (absent to very strong). For the pleasantness and unpleasantness dimensions, the arousal SAM figures were employed to ensure maximum comparability between the scale types.

The order of bipolar and unipolar scales, of arousal-valence and of pleasantness-unpleasantness dimensions on each page of the rating booklets, as well as the general scale order, were randomized and counterbalanced across participants. Thus, ratings on both unipolar and bipolar scales were obtained from the same population, allowing us to directly relate mean ratings to each other. Rating booklets were available in German as well as English (see “Participants” for details). Distribution of different booklet versions in each language was approximately equal (see Additional file 2,.pdf). The layout of one example booklet in English is accessible as an additional.pdf file (see Additional file 3).

### Participants

A total of 278 undergraduate students took part in this experiment in the language (English or German) of their degree program. Only participants with normal or corrected-to-normal vision were; 35 were excluded (9 after stating they had uncorrected vision impairments, 26 due to missing information regarding vision impairments). The data of one participant who had accidentally received a booklet in the wrong language was also excluded from our analyses. In the end, the data of 242 participants (*M*_*age*_=21.35, *SD*=3.38) were analyzed (see Table 1 for population characteristics).

**Table 1:** **Population characteristics**

		**N**	**% male** ^**1**^
Total		242	26.67
Handedness	Right	208	27.18
	Left	21	19.05
	Both	7	42.86
	Missing	6	50.00
Citizenship	German	197	22.34
	Other	31	37.50
	Missing	14	57.14
Native language(s) ^2^	German	192	23.40
	English	7	28.57
	Only other	29	41.38
	Missing	14	28.57
Field of study ^3^	Alternative tourism (G)	31	16.67
	Education (G)	81	11.11
	International relations (E)	78	35.90
	International business (E)	40	42.50
	Other (6 E, 3G)	9	44.44
	Missing (1E, 2G)	3	33.33
Booklet language	English	125	39.20
	German	117	13.04
Scale order	First bipolar	118	23.73
	First unipolar	124	29.51

^1^gender information was not provided by two participants.

^2^participants could indicate more than one native language.

^3^letters in brackets indicate booklet language, E = English, G = German.

### Design and procedure

Our study was conducted during regular university classes by a trained experimenter in the course’s language of instruction (see Table 1 for a list of course programs and languages). Participants were informed that their participation was voluntary and could be discontinued at any time.

Each session was subdivided into different sections (see Figure 1C for an overview of the reported study’s procedure). Participants were given detailed oral instructions and were also referred to the written instructions in each testing booklet. They were asked to rate 84 pictures according to their own emotional experience using the 9-point scales described above (see Additional file 1 for the pseudo randomized order of picture presentation, the stimuli shown first in the gender attribution task and repeated during the rating procedure). The type of rating scale used was changed halfway through the set of pictures.

For the arousal and bipolar valence rating scales, instructions were adapted from the original IAPS rating procedure (Lang et al. 1997b). Instructions for the unipolar pleasantness and unpleasantness rating scales were adapted from the bipolar rating scales’ instructions. Pictures were projected in regular lecture halls. The tests followed the IAPS protocol (Lang et al. 1997b), but rating time was shortened to 10 s (as participants had to rate only two of three dimensions; see Figure 1B). The order of picture presentation was pseudo randomized - and neither the variations of the same stimulus category (need-of-help, child/adult, child/bird), nor of the concrete situations (e.g. opening a door) were ever repeated directly after each other. The existence of two different picture subsets (“need-of-help” and “social context”) was not apparent to the participants.

### Data analyses

Data analyses were conducted using the GNU software R (version 3.0.2). Arousal, pleasantness and unpleasantness ratings were coded from 0 (calm, no pleasant feelings, no unpleasant feelings) to 8 (excited, strong un/pleasant feelings), bipolar valence ratings from −4 (unhappy) to 4 (happy). For each picture, we calculated mean ratings for all four dimensions across participants, and also for men and women separately. Each picture received a minimum of 106 valid ratings for each dimension (see Additional file 1 for exact *n*s), ratings were considered invalid if multiple or no responses were given for one picture. Ratings were averaged per picture across all booklet versions (see Additional file 2 for distribution and impact of booklet versions and language on average ratings).

All analyses were based on effect sizes and estimation of confidence intervals. As the use of null-hypotheses testing has been shown to facilitate arbitrary and possibly misleading results (see e.g. Cumming 2014; Kline 2004), we refrained from performing traditional p-value calculations. Interpretation of CIs can be related to classical *p*-values in the following way: non-overlapping CIs of two estimated mean values indicate a meaningful difference between these two. However, the approach is not equivalent to null-hypothesis testing in that non-overlapping CIs do not indicate non-significance. Data interpretation based on CIs can be considered more conservative as null-hypothesis testing while having the advantage of accurately conveying the uncertainty of findings instead of dichotomizing them into “significant” and “non-significant”.

To assess picture content effects, mean ratings and 95% CIs surrounding the estimated mean were calculated using the R functions CI and group. CI from the library Rmisc. Cohen’s *d* was used as an estimate of effect size using the function cohen.d from the R package effsize. In order to correct for biased population estimation of Cohen’s *d*, Hedges’ correction was used (Hedges and Olkin 1985). Magnitude of Cohen’s *d* was judged based on suggestions by Cohen (Cohen 1969). As men and women have been shown to differ regarding various aspects of emotional experience (Bradley et al. 2001; Brody and Hall 2008; Fischer 2000), means and CIs were also calculated for men and women separately. Thus, all comparisons, between picture categories as well as between men and women, were based on mean ratings per picture and hence equal sample sizes.

The relation between average ratings on the different scales and dimensions was assessed with linear models. The only predictor used was the rating on the scale to which the relation was to be determined. Additionally, Pearson correlations were calculated for linear relations. In order to estimate the amount of variance in ratings explained by relations to another scale, we used a split-half procedure and computed-squared correlations based on item analysis within one scale (“reliability” scores). This “reliability” score served as the maximum amount of variance possibly explained by another variable. The total explained variance was hence defined as adjusted *R*^2^ divided by this “reliability” value.

Pre-analyses showed that the pictures’ sequence position did not influence ratings on any of the four dimensions, all |*r*|(82)≤0.19. Also, the gender of the depicted child did not affect ratings on either dimension, the largest, but still not meaningful difference emerging in valence ratings with *M*_*boys*_=−0.67, [ −1.08, −0.26], and *M*_*girls*_=−0.86, [ −1.26, −0.45]. No interaction of the gender of the depicted child and that of the participant (own-gender effect) emerged; the largest, but again not meaningful difference between boy and girl depictions was present for men’s valence ratings, *M*_*boys*_=−0.48, [0.04, −1.00], and *M*_*girls*_=−0.81, [ −0.34, −1.29]. Further analyses were conducted across presentation sequence and gender of the depicted child. The language only influenced mean ratings in that arousal ratings were a little higher and unpleasantness ratings a little lower for participants who received English rather than German material and instructions. Importantly, the language never affected picture content effects. Because of the negligible importance of these findings to our current research question, the results of the analyses of language differences can be found in the Supplementary Online Material (see Additional file 2).

## Results

### “Need-of-help” stimulus subset

#### Need-of-help is rated unhappier and more arousing

**Figure 2:**
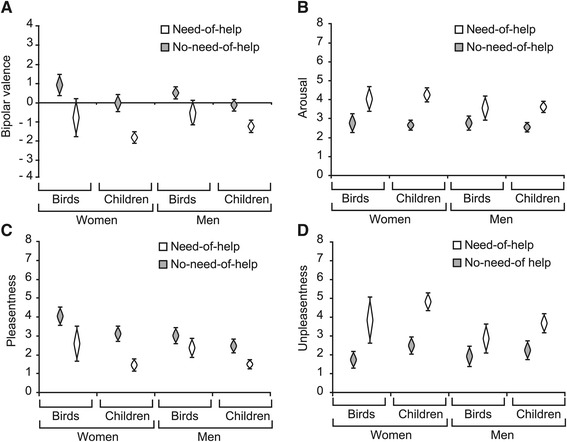
Effects of need-of-help on bipolar valence **(A)**, arousal **(B)**, pleasantness **(C)** and unpleasantness **(D)**. Means are shown separately for pictures of children and birds as well as for women (left side of each graph) and men (right side of each graph). White cat’s eyes represent means for need-of-help pictures, gray ones represent means for no-need-of-help pictures. The length of cat’s eyes indicates 95% confidence intervals.

Mean ratings and according 95% CIs for each picture category are provided in Table 2. Regarding bipolar valence ratings (unhappy to happy through neutral) we found that need-of-help pictures were rated considerably less happy than no-need-of-help pictures, *d*=−1.60. This effect was large for men and women, as well as for pictures of children and birds (see Figure 2A), all *d*≤−1.43. Furthermore, pictures of birds were rated higher in valence than pictures of children (see Figure 2A and Table 2), *d*=0.78, albeit this effect was smaller than the one of need-of-help content. A higher valence rating in the case of bird compared to human depictions meant a mean valence of nearly zero and thus close to neutral. Male and female participants did not differ in their mean valence ratings, as indicated by largely overlapping CIs (see last rows of Table 2).

**Table 2:** **Mean ratings per picture category and gender for the need-of-help subset**

		***M***			
		**(Lower 95% CI / Upper 95% CI)**			
**Comparison**	**Group**	**Bipolar valence**	**Arousal**	**Pleasantness**	**Unpleasantness**
Need − No need	Need	**−1.19**	**3.89**	**1.87**	**3.92**
		(−1.45 / −0.92)	(3.67 / 4.11)	(1.62 / 2.11)	(3.56 / 4.28)
	No Need	**0.24**	**2.68**	**3.08**	**2.16**
		(0.02 / 0.46)	(2.54 / 2.83)	(2.84 / 3.32)	(1.92 / 2.40)
Bird − child	Bird	**0.06**	3.29	**3.02**	**2.59**
		(−0.32 / 0.43)	(3.00 / 3.59)	(2.68 / 3.36)	(2.15 / 3.03)
	Child	**−0.79**	3.28	**2.15**	**3.31**
		(−1.03 / −0.54)	(3.07 / 3.50)	(1.92 / 2.38)	(2.97 / 3.64)
Women − men	Women	−0.52	3.44	2.66	3.34
		(−0.89 / −0.15)	(3.17 / 3.72)	(2.32 / 3.01)	(2.90 / 3.77)
	Men	-0.41	3.13	2.27	2.75
		(-0.66 / -0.17)	(2.92 / 3.34)	(2.05 / 2.49)	(2.43 / 3.07)

Compared means whose 95% CIs do not overlap are highlighted in bold.

Regarding arousal ratings, need-of-help depictions were rated as more arousing than no-need-of-help depictions (see Figure 2B) with a similarly large effect size as for valence ratings, *d*=1.80. The gender of participants only influenced arousal ratings for one picture category: Women rated need-of-help pictures to be moderately more arousing than men did, *d*=0.75. All remaining comparisons between pictures of diverging content and between male and female participants exhibited overlapping 95% CIs around the estimated means, indicating that there were no meaningful differences.

#### Need-of-help is rated less pleasant and more unpleasant

For pleasantness ratings, the largest effect was again the one of need-of-help: As is to be expected, need-of-help pictures were rated less pleasant than corresponding no-need-of-help pictures (see Figure 2C and Table 2, column 3), *d*=−1.37. Also, pictures of birds were rated as more pleasant than pictures of children, *d*=0.88. Again, need-of-help content had the same effect on men’s and women’s ratings (see Figure 2C). Men and women differed only in their pleasantness ratings of no-need-of-help pictures of children and birds, with women giving higher pleasantness ratings, *d*=0.95.

For unpleasantness ratings, need-of-help had the strongest effect, as was the case for all other rating dimensions. Need-of-help depictions were rated more unpleasant than no-need-of-help depictions, *d*=1.57, (Figure 2D and Table 2). Pictures of children were rated as more unpleasant than pictures of birds to a medium extent, *d*=0.52. Again, CIs for mean unpleasantness ratings of men and women overlapped considerably, indicating that overall these were not different from one another and the impact of need-of-help content’s effect was approximately the same for men and women (see Figure 2D). However, complementary to pleasantness ratings and similar to bipolar valence ratings, a specific difference between men’s and women’s ratings was evident for need-of-help pictures: Women rated need-of-help pictures to be more unpleasant than men, *d*=0.89.

### “Social context” stimulus subset

#### Differential effects of social context on valence and arousal ratings

The “social context” subset served to evaluate whether seeing the act of (simple, everyday) helping influences emotional ratings over and above the effects of seeing someone who doesn’t need help. We compared the emotional ratings of pictures showing an adult helping a child (“social helping”) to those of pictures showing a passive adult bystander (“social passive”) - in both cases the need-of-help was resolved. Control pictures of the same child (“child-alone”) and of the same adult (“adult-alone”) as in the ocial-passive” category allowed us to assess the influence of social context. Mean ratings for all picture categories, as well as for men and women, are given in Table 3.

**Table 3:** **Mean ratings per picture category and gender for the social context subset**

	***d***			
	**(Lower 95% CI / Upper 95% CI)**			
	**Bipolar valence**	**Arousal**	**Pleasantness**	**Unpleasantness**
Child-alone	−0.02	2.70 ^2^	2.99	2.44
	(−0.66 / 0.63)	(2.34 / 3.07)	(2.47 / 3.51)	(1.76 / 3.11)
Adult-alone	0.10	1.45	2.60	1.80
	(−0.52 / 0.72)	(1.27 / 1.62)	(2.08 / 3.13)	(1.22 / 2.39)
Social passive	0.55	2.32 ^2^	3.81 ^1,2^	1.74
	(0.00 / 1.10)	(2.04 / 2.61)	(3.29 / 4.33)	(1.24 / 2.24)
Social helping	0.99	2.40 ^2^	4.23 ^1,2^	1.56
	(0.50 / 1.48)	(2.15 / 2.64)	(3.66 / 4.80)	(1.14 / 1.98)
Women	0.46	2.17	3.56	1.89
	(0.15 / 0.77)	(1.97 / 2.38)	(3.23 / 3.90)	(1.61 / 2.16)
Men	0.24	2.33	2.97	1.87
	(0.03 / 0.45)	(2.51 / 2.15)	(2.68 / 3.25)	(1.61 / 2.12)

^1^CI not overlapping with “Child-alone” pictures, indicating a meaningful difference.

^2^CI not overlapping with “Adult-alone” pictures, indicating a meaningful difference.

No systematic differences were observed between valence ratings to the “child-alone” and “adult-alone” pictures, nor between the “social helping and the “social passive” categories (see Table 3). The same was true for general and social context specific differences in bipolar valence ratings between men and women.

**Figure 3:**
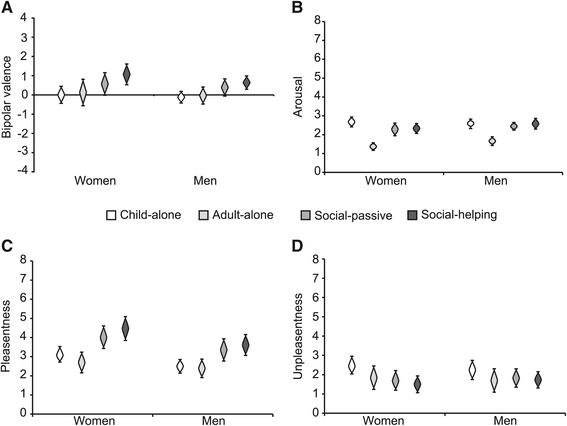
Effects of social contexts on bipolar valence **(A)**, arousal **(B)** pleasantness **(C)** and unpleasantness ratings **(D)**. White cat’s eyes represent ratings for “child-alone” pictures, light gray ones ratings for “adult-alone” pictures, middle gray ones ratings for “social-passive” and dark gray ones ratings for “social-helping” pictures. The length of cat’s eyes indicates 95% confidence intervals.

Arousal ratings were considerably lower for “adult alone” pictures than for the other categories (Figure 3B), all *d*≥2.30. These differences were evident for men and women alike. No general difference between men’s and women’s arousal ratings was evident. One difference between arousal and bipolar valence ratings was that the CIs of arousal ratings were narrower than those of bipolar valence ratings (compare Figure 3A and 3B and first and second column of Table 3), reflecting stronger homogeneity in arousal ratings than in valence ratings. When interpreting these effects one should keep in mind that they were stable and consistent, even though arousal ratings across all picture categories remained low in absolute magnitude, as is to be expected for relatively harmless depictions of trivial situations.

#### Social context increases pleasantness, but does not affect unpleasantness ratings

Pleasantness ratings were higher for both social context picture categories (“social-helping” and “social passive”) than for pictures of individual persons (see Figure 3C), all *d*≥0.99, an effect not observed for overall valence ratings. Moreover, pleasantness ratings for this “social context” subset of pictures tended to be higher for women than for men, *d*=0.61, as indicated by nearly non-overlapping CIs, *M*=3.56, 95% CI [3.23, 3. 90], for women, and *M*=2.97, [2.68, 3.25], for men.

Unpleasantness ratings were slightly higher for pictures that showed the child alone than for “social helping” pictures, when data for men and women was considered separately (see Figure 3D), *d*=−0.89. Moreover, unipolar unpleasantness ratings, unlike unipolar pleasantness ratings, were not influenced by the gender of participants.

### Summary of picture content’s effects

Visual stimuli showing someone in need-of-help in a simple, non-dramatic everyday situations were rated lower in valence and higher in arousal (see Figure 2A and 2B), less pleasant and more unpleasant (see Figure 2C and 2D) than those showing somebody reaching a goal. Need-of-help effects were identical for pictures of birds and children, as well as for male and female participants. The depiction of a bird altered self-reported emotional experiences of un-/pleasant feelings to a lesser degree, similarly to the alterations observed for bipolar valence: pictures of birds were rated as somewhat more pleasant, less unpleasant and higher in valence than pictures of children, no effects of bird-depiction were observed for arousal. Using different scales to assess valence (bipolar valence versus intensity of pleasantness and unpleasantness) led to diverging results regarding differences according to social context: There was no indication for effects on valence, while pleasantness ratings were higher for social-context pictures than for those showing a child or an adult alone. Picture content influenced men’s and women’s self-reported emotions in very similar ways, with a slight tendency for women to rate depictions of need-of-help as more arousing, less pleasant and more unpleasant than men.

### Relation between different rating dimensions

**Figure 4:**
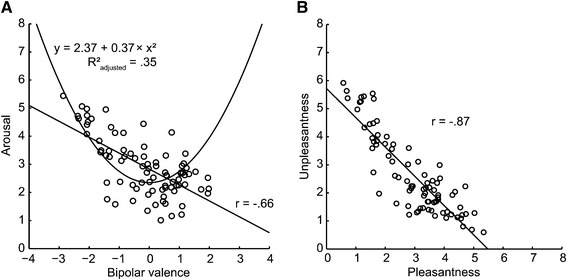
Relation between bipolar valence and arousal **(A)** and between pleasantness and unpleasantness ratings **(B)**. Each data point corresponds to mean ratings for one picture. Regression lines were calculated using MatLab LSD.

The relation between arousal and valence dimensions for each picture across the stimulus set is illustrated in Figure 4A. In accordance with the literature that often includes a much broader range of valence and arousal ratings, we found a quadratic relation between bipolar valence and arousal ratings, *r*(82)=.60, 95% CI [.44,.72], $R^{2}_{\textit {adjusted}} = 0.35$. However, in our study the linear relation between those scales was just as strong, *r*(82)=−.66, [-.77, -.52], and accounted for 43% of the variance. Ratings of pleasantness and unpleasantness showed a strong negative linear correlation, *r*(82)=−.87, 95% CI [-.92, -.81], $R^{2}_{\textit {adjusted}} = 0.76$, illustrating the antagonistic relation between both rating scales (see Figure 4B).

**Figure 5:**
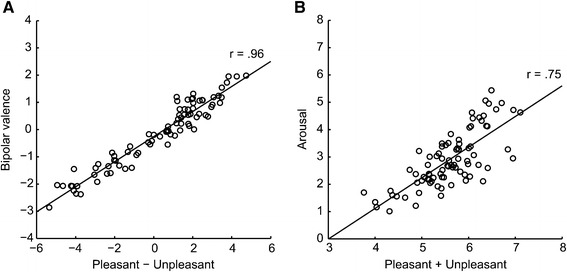
Relation between aggregated unipolar pleasantness and unpleasantness ratings and bipolar valence **(A)** as well as arousal **(B)**. Each data point corresponds to mean values for one picture. Regression lines were created using MatLab LSD.

All participants used arousal and bipolar valence scales for one half of the stimuli, pleasantness and unpleasantness scales for the other half. This within-subject design allowed us to directly relate the two types of ratings to each other. First, we investigated whether bipolar valence can be inferred from the difference between pleasantness and unpleasantness ratings (see Kron et al. 2013). The correlation between this difference and bipolar valence ratings was nearly perfect (see Figure 5A), *r*(82)=.96, 95% CI [.94,.98], suggesting that participants employed the two types of scales similarly. If considering the “reliability” of the valence scale, 99% of the variance in valence ratings could be inferred from the difference between pleasantness and unpleasantness ratings, indicating that the information inherent in bipolar valence ratings can be derived almost completely by calculating the difference between unipolar pleasantness and unpleasantness ratings. Analyses of co-occurring pleasantness and unpleasantness ratings above zero, so called mixed feelings, indicated that pleasantness and unpleasantness rating scales can potentially provide information not accessible through valence scales (see Additional file 2 for analyses regarding mixed feelings).

We also tested whether the overall intensity of pleasant and unpleasant feelings (defined as the sum of both ratings per picture) could account for arousal ratings, as has been demonstrated for IAPS-Stimuli before (Kron et al. 2013). The mean sum of pleasantness and unpleasantness ratings accounted for 56% of variance in averaged arousal ratings (see Figure 5B) and showed a high correlation with arousal ratings, *r*(82)=.75, 95% CI [.64,.83]. Using the same split-half reliability procedure as described for valence ratings, 65% of variance in arousal ratings was explained by this relation.

### Aggregated unipolar pleasantness and unpleasantness ratings can replicate effects on bipolar valence and arousal scales

So far, we have demonstrated that there is a close relation between aggregated unipolar pleasantness and unpleasantness ratings and ratings of arousal and bipolar valence (see Figure 4). The sum of pleasantness and unpleasantness ratings can be regarded as an indication of emotional experience’s overall intensity and seems to relate to arousal ratings. The difference between pleasantness and unpleasantness ratings correlates almost perfectly with valence ratings, indicating that valence ratings on a bipolar scale reflect combined pleasant minus unpleasant experiences. Moreover, we have shown how help-related picture content and social context variations change emotional ratings of bipolar valence and arousal as well as of unipolar pleasantness and unpleasantness. A remaining question is whether results regarding both need-of-help and social content analyses for aggregated pleasantness and unpleasantness ratings can mirror content related results for ratings on bipolar valence and arousal scales. Our study is well suited to answer this question, as ratings were collected within the same population using both scale types.

**Figure 6:**
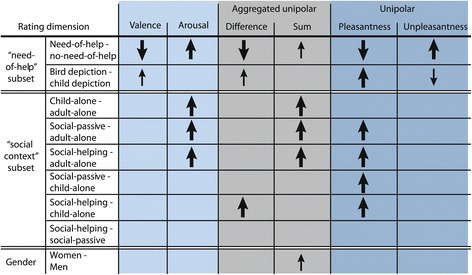
Overview of effect sizes on all dimensions analyzed. Effect sizes (Cohen’s *d* with Hedge’s correction) symbolized by arrows are shown for bipolar valence and arousal (left, light blue), unipolar pleasantness and unpleasantness (right, dark blue) and aggregated unipolar ratings (middle, gray) separately. Arrows’ appearance illustrates magnitude of effect sizes with bold = large (*d*>.80); 0^∘^ vertical = medium (*d*>.50). Arrows are shown only for *d*s with CIs not overlapping 0. Note that differences were the same for bipolar valence/arousal and aggregated unipolar ratings with the exception of the gender main effect and the comparisons involving “social-passive” pictures.

Thus, we repeated analyses of the “need-of-help” and the “social content” subsets for the difference between as well as the sum of pleasantness and unpleasantness ratings. When we considered picture content, the same pattern of effects emerged for the sum of pleasantness and unpleasantness ratings and for arousal ratings, as well as for the difference between pleasantness and unpleasantness ratings and bipolar valence ratings (see Figure 6). The detailed effect sizes for both picture subsets concerning aggregated pleasantness and unpleasantness ratings can be found in Additional file 2.

There were only two small differences evident for the results obtained on valence and arousal scales compared to the difference and the sum of pleasantness and unpleasantness ratings. Gender differences were more pronounced when we considered the sum of pleasantness and unpleasantness ratings rather than arousal ratings alone: Women’s sum score was larger than men’s, *d*=1.19, and *d*=0.69, for the “need-of-help’ and “social context” subsets respectively. Perhaps the summation of two ratings amplified smaller differences in each of the summands. There was one other small incongruence between content effects observed for bipolar valence ratings and the difference between pleasantness and unpleasantness ratings: “social helping” pictures received a higher aggregated score than “child-alone” pictures, but no lower valence ratings. It should be noted, however, that when we distinguished between men’s and women’s bipolar valence ratings, a tendency for higher valence ratings for “social helping” compared to “child-alone” pictures reached a considerable effect size, *d*=1.17. Differences between “child-alone” and “social helping” pictures hence cannot be considered robust and are probably too uncertain to be reliably detected. Therefore, we believe that this divergence in result pattern does not forbid the overall conclusion that effects observable on bipolar valence ratings could be replicated using the difference between pleasantness and unpleasantness ratings. The same was true for arousal ratings and the sum of pleasantness and unpleasantness ratings with the exception of gender differences.

## Discussion

We assessed young adults’ subjective emotional experiences elicited by depictions of trivial need-of-help situations and variations of social context. In order to also investigate how the results obtained with bipolar and unipolar scales relate to each other, we employed two types of rating scales in a within-subject design. Pictures showing someone needing help to reach a basic goal were rated lower in valence, more arousing, less pleasant and more unpleasant than almost identical pictures showing someone achieving a goal. All effects of need-of-help content were consistent and large; they were not restricted to humans and realistic needs, but extended to humanized depictions of birds in situations untypical for their species.

Our findings confirm the common but previously untested assumption that negative affect is elicited even by trivial need-of-help and is probably a core component of an empathic emotional response (Cao 2010; Eisenberg et al. 1989; Fabes et al. 1993; Rameson et al. 2012; Stocks et al. 2009; Sze et al. 2012). The fact that the stimuli were relatively neutral black-and-white drawings and that we deliberately abstained from depicting dramatic or dangerous need-of-help situations strengthens the assumption that everyday need-of-help is more likely to elicit negative emotions than similar neutral situations where there is no need-of-help. In this way, the present results expand the knowledge of affective processing by demonstrating that the content dimension need-of-help is a relevant aspect in eliciting negative subjective emotions. Heightened arousal, or overall higher level of feelings’ intensity, elicited by trivial need-of-help (see Figure 5A) might be related to a higher preparedness for initiating an action, e.g. to help the needy person, mirroring the assumptions of the aversive arousal theory (Dovidio 1991; Piliavin et al. 1982). Higher self-reported arousal could also relate to more basic attention processes as discussed in previous studies on attentional biases and affective processing (Keil et al. 2003; Lang et al. 1997a). Since in this study we only assessed subjective reports of emotional experience and did not directly relate those to real-life helping behavior or to psychophysiological measures, the plausible link of these findings to actual helping behavior remains to be explored in future research.

Besides looking at the effects elicited by need-of-help itself, the present study also assessed whether differential responses to active helping compared to a passive social situations will be reported, even if the need-of-help was resolved in both conditions. This question is particularly important because previous research has failed to specify whether active helping elicits positive affective response beyond the resolved need itself. We found no evidence that seeing somebody helping differentially affected self-reported emotional experiences compared to observing an identical social situation without need-of-help. Thus, we conclude that in the case of relatively harmless depictions of need, and thus relatively minor necessary action, the active prosocial involvement itself does not add substantially to the elicited affective response.

Next, we asked whether depictions of a social interaction, in this case an adult and a child shown together in the absence of need-of-help, would be rated differently from if each agent was shown alone. To our knowledge, this is the first study to investigate the differential effects of a situation’s social context on self-reported emotional experience. Even though some research has shown that socially relevant stimuli produce discernible physiological (Britton et al. 2006a) and neural responses (Britton et al. 2006b; Sakaki et al. 2012) by comparison to completely non-social stimuli (scenes not involving humans), the question of whether comparable pictures showing two interacting persons rather than one human elicit different emotional experiences has not so far been addressed. The strongest effect we found with regard to social context variations was that “adult-alone” pictures were rated lowest in arousal (see Figure 3B) compared to all other picture categories. The underlying reasons for this effect are unclear and were not the subject of the reported investigation. One possible explanation would be that the depiction of an action, present for pictures of children alone and for the two social context categories alike, might have increased perceived arousal. The “action” aspect in this study was, however, confounded with the presence of a child, so it is also possible that the higher arousal ratings relate to the depictions of children. The depiction of an adult and a child together increased pleasantness ratings (see Figure 3C). Higher pleasantness ratings found for pictures showing two rather than one person might be an indication for a social context preference. Alternatively, the depiction of a child interacting with an adult might have led to specific self-reported emotions. However, the depiction of an adult and child together scarcely affected bipolar valence ratings. Results on unipolar and bipolar valence scales differed in their interpretation with regard to the effect of social context. This divergence exemplifies that assessing bipolar valence alone may draw a different picture of stimulus content’s influences on positive and negative affect than pleasantness and unpleasantness ratings. This divergence does not stand in contrast to the finding that an aggregated score derived from both pleasantness and unpleasantness ratings replicates effects on bipolar valence scales. On the contrary, such an aggregated score represents the same simplification of pleasantness and unpleasantness scores as bipolar valence scales that does not allow distinction of effects on positive versus negative affect.

An additional aim of this study was to re-evaluate the relation between ratings provided on two different types of scales for self-reported emotional experience: arousal (calm to excited) and bipolar valence scales (unhappy to happy), compared to unipolar scales, assessing the intensity of pleasant and unpleasant feelings from absent to strong. Results of our study regarding the inter-relation between ratings obtained with these two types of scales are highly similar to previous findings (Ito et al. 1998; Kron et al. 2013). This was the case even though our visual stimuli are more abstract and show trivial, non-dramatic everyday situations only, in contrast to the IAPS stimuli used before that intentionally contain extremely pleasant and unpleasant photographic images. The difference between unipolar pleasantness and unpleasantness ratings (see Figure 5A) explained 99% of variance in mean bipolar valence ratings. For arousal ratings, 65% of variance could be explained by the overall intensity of unipolar pleasantness and unpleasantness ratings, i.e. their sum (see Figure 5B). This generalization of close mapping of pleasantness and unpleasantness to arousal and particularly to bipolar valence ratings provides evidence for the robustness of previous findings (Ito et al. 1998; Kron et al. 2013). Even though in psychological research a 65% of variance explained is considered high and consistent, aggregated pleasantness and unpleasantness ratings seem to reflect valence ratings to a higher degree, than arousal ratings. This finding contributes to an ongoing discussion about the concept of arousal and its relation to the valence dimension (Kron et al. 2013). Intercultural research adds to the notion that subjective understanding of the arousal concept vary with language and culture (Deák et al. 2010; Dufey Domínguez et al. 2011; Grühn and Scheibe 2008; Lasaitis et al. 2008; Ribeiro et al. 2005; Schmidtke et al. 2014).

Our findings do not contradict the notion that physiological and neuronal responses can be attributed to the distinct, possibly orthogonal dimensions of valence and arousal (Anders et al. 2004; Bradley and Lang 1994, 2000; Wilson-Mendenhall et al. 2013). What the present results suggest is that even though physiological responses distinguish between arousal and valence, people’s self-reports of emotional experience might be measured by means of positive and negative affect that do not require an explicit report of arousal independent form valence. As was pointed out earlier, findings from self-reports need not be congruent with the physiological ones underlying them (Barrett et al. 2009). An additional advantage of the unipolar pleasantness and unpleasantness is the assessment of mixed feeling, an aspect that seems to culminate in stimuli classified as rather neutral on the bipolar valence scale (Hemenover and Schimmack 2007; Kreibig et al. 2013; Kron et al. 2013; Larsen and Green 2013; Schimmack et al. 2001; Schimmack and Colcombe 2007).

Despite some differences, the strong correspondence of content-related effects provides further evidence for the congruence between aggregated unipolar and arousal as well as bipolar valence ratings. The same effects of need-of-help, bird depiction, as well as social context emerged regarding the sum of pleasantness and unpleasantness ratings compared to arousal ratings and regarding the difference between pleasantness and unpleasantness compared to bipolar valence ratings (see Figure 6). Results, however, diverged regarding two aspects: First, the difference between “child-alone” and “social-passive” pictures was not meaningful for bipolar valence ratings across participants, whereas the difference between pleasantness and unpleasantness ratings was higher for “social-passive” than for “child alone” pictures. However, if interpretation is adequately based on the complete CIs range, this divergence can be considered minor in impact. Furthermore, whereas gender differences were small across content categories for arousal ratings, meaningful gender differences were evident regarding the sum of pleasantness and unpleasantness ratings. This difference could have arisen partly from the aggregation of pleasantness and unpleasantness scores that doubles up gender’s otherwise small effects on valence ratings. This effect may arise from women’s tendency to report higher emotional intensity (e.g. Fujita et al. 1991; Lang et al. 1993). Overall, we did not find strong indications for general rating tendencies specific to men and women: For both picture subsets the gender difference only once proved to lie meaningfully above zero, i.e. with regard to pleasantness ratings for the “social context” subset. In all cases, the magnitude of gender differences was overridden by the effects of help-related picture content (see Tables 2 and 3). The small magnitude of gender differences is in line with findings regarding adults’ ratings of pictures moderate in emotional intensity (McManis et al. 2001). The small occasional differences reported here neither clearly support, nor completely contradict these earlier findings. A potential explanation is that our stimuli were designed to elicit feelings of low to moderate intensity and that gender differences in emotional experience emerge more clearly for more extreme stimuli.

### Limitations and future directions

The study reported here has assessed participants’ self-reported emotional experiences elicited by help-related and social picture content in a controlled but therefore partially limited fashion. In contrast to many previous studies we used comic-like pictures instead of more naturalistic stimuli, such as photographs or real-life sounds. We show that the effects of (social) picture content can be studied using highly controlled pictures that are very similar with regard to all but the relevant dimensions (content, perceptual properties, etc.). Some recent findings suggest that non-photorealistic images elicit muted emotional responses compared to realistic photographs (Mould et al. 2012). Still, the effect sizes reported here were considerable. It might thus be advisable to implement minimal-change comparisons like we did in studies with real-life photographs and videos that would have to be staged or adapted from real life situations. This combination would prevent results from being confounded with a-priori differences in stimulus properties between conditions, such as luminance, contrast, complexity, etc., while increasing ecological validity in comparison to the stimuli used in this study.

An objection to the suitability of our stimuli might be that their ratings are not comparable to those of previous studies using stimuli such as the IAPS, because of the lower overall magnitude of valence and arousal expected for our relatively neutral stimuli. Nonetheless, there is evidence that the ratings obtained for our stimuli do not stand at odds with other standardized stimulus sets. In studies that select pictures within a category relying on the valence dimension only, mean arousal ratings found for stimuli in the “pleasant” category have in many cases been similar to ours (see e.g. Table one in Grühn and Scheibe 2008). We found only a weak quadratic relation between valence and arousal ratings. The linear relation between valence and arousal was similarly strong in our study, showing a negative correlation between the two dimensions. This has been found previously in numerous studies, involving participants other than North-Americans (Deák et al. 2010; Dufey Domínguez et al. 2011; Grühn and Scheibe 2008; Lasaitis et al. 2008; Ribeiro et al. 2005; Schmidtke et al. 2014), putting forward the explanation that in some languages (and/or cultures) the term arousal is more likely to be linked with unpleasant experiences than with pleasant ones. In line with these studies, the more pleasant pictures in our study were not rated as being much higher in arousal than the neutral ones, whereas unpleasant ones were (see Figure 3). This weakened the quadratic relation but strengthened the linear relation between valence and arousal ratings compared to the IAPS ratings of North American observers. In future, it will be necessary to bring together evidence from multiple studies with more diverse populations to understand the structure of self-reported emotional experiences particularly with regard to the arousal dimension.

The bird depictions added a further categorical dimension to our stimulus set that was unrelated to help content. The high level of perceptual correspondence and the depictions of identical situations in the bird and child pictures ensured that divergence in results between those categories may not stem from differing situational content or lower-level perceptual picture properties. A limitation to this approach is, however, that we put birds in situations not adequate for their species and thus might have elicited greater uncertainty with regard to the extent the birds really needed help. Thus, the present study is not suited to resolving the question of whether or not empathic responses are comparable for human and non-human agents. For this purpose, a different set of stimuli that fulfilled the criterion of comparability regarding all three picture characteristics would be necessary: perceptual properties, situational content and situational adequacy.

Also, the effects reported here may hold true for the undergraduate population assessed, but not necessarily for other age-, profession- or cultural groups. Emotional ratings have been shown to undergo changes throughout a lifespan (Backs et al. 2005; Grühn and Scheibe 2008) just as empathic concern does (Sze et al. 2012). The assessment of how children’s response to help-related content develops with age is a particularly fruitful approach towards understanding how emotional responses shape social cognition. The gender differences reported in this study have to be interpreted with caution, as gender schemas were probably salient for our participants who were required to make gender attributions in the preceding experiment. Thus, it is possible that the relatively minor gender differences reported here were greater than those of other studies not involving tasks related to gender attributions.

Another limitation of our study is that we focused on self-report measures only. However, it is plausible that dissociation between arousal and valence to help-related picture content can be found in physiological responses. The highly standardized and unambiguous visual stimuli we used are well suited for psychophysiological and neuroimaging experiments. Moreover, we measured emotional response to need-of-help depiction and identified specific effects on core emotions, but we did not relate these to any personality characteristics or real-life helping behavior. Psychophysiological and behavioral studies that tackle the above themes will provide interesting directions of research in future.

## Conclusion

In conclusion, our study provides insights into people’s emotional experiences elicited by help-related content on two levels. In a content-related perspective, seeing someone in need-of-help has meaningful and strong effects on self-reported emotional experiences, independent of the rating scale used. This is the case, even when the need-of-help is depicted by comic-like drawings showing harmless, everyday situations. Depictions of (basic) active helping had no discernible effect on any rating dimension used in this study. The large and generalizable influence of help-related content on self-reported emotional experiences emphasizes the importance and automaticity of experiencing emotions congruent with those presumably experienced by others. From a methodological perspective, bipolar valence and arousal ratings can be inferred to large degrees from aggregated unipolar pleasantness and unpleasantness ratings for relatively neutral pictures - just as has been demonstrated for IAPS pictures. The correspondence for valence is almost perfect, whereas for the arousal dimension it is still strong, but lower. This finding raises the question of what aspects of self-reported arousal can be considered distinct from the overall intensity of subjective emotions expressed by pleasant and unpleasant feelings.

## Availability of supporting data

The data set supporting the results of this article is available in the Open Science Framework repository, https://osf.io/r2tep/.

## Additional files

Additional file 1
**Stimulus material.** Complete list of all stimuli used including thumbnails, names, trial number, mean bipolar valence, arousal, pleasantness, unpleasantness, and mean intensity of mixed feelings for each picture.

Additional file 2
**Additional results and analyses.** Lists the detailed number of booklets for each version and language analyzed as well as magnitude of all effects on emotional ratings. Table listing the detailed effect sizes and CIs for the analyses of help-related content regarding the difference between and the sum of pleasantness and unpleasantness ratings. Analyses of mixed feelings. Figure illustrating scoring procedure. Figure displaying means per picture on each scale type colored by picture content category.

Additional file 3
**Example rating booklet exert.** Exemplary instruction and first rating pages for one of the English rating booklets versions.
