# Supplementary material for: Does it matter how you ask? Self-reported emotions to depictions of need-of-help and social context
Source: BMC Psychol. 2015 Apr 7;3(1):10. doi: 10.1186/s40359-015-0066-3 (PMC4403975; doi:10.1186/s40359-015-0066-3)
Supplement: Additional file 2 — Additional results and analyses. Lists the detailed number of booklets for each version and language analyzed as well as magnitude of all effects on emotional ratings. Table listing the detailed effect sizes and CIs for the analyses of help-related content regarding the difference between and the sum of pleasantness and unpleasantness ratings. Analyses of mixed feelings. Figure illustrating scoring procedure. Figure displaying means per picture on each scale type colored by picture content category. [file 40359_2015_66_MOESM2_ESM.pdf]

Supplementary Material - Does it matter how you ask? Self-reported emotions to depictions of need-of-help and social context

Additional results and analyses

Influences of rating material on mean ratings

We used four different booklets, which were all available in both English and German to correspond with the language of the course taken by the student participants. The four versions allowed us to counterbalance the effects of scale order (rst bipolar scales or rst unipolar scales) and of dimension order (bipolar valence/pleasantness as rst dimension on the top of the page or arousal/unpleasantness). As different versions were used in nearly equal numbers (see Table S1), averaging across versions for all analyses presented in the main paper ensures that the effects of other analyses were not confounded with effects stemming from the booklet itself.

Table S1. Number of different booklet versions used in each language.

		Language	
Scale order	Dimension order	English	German
First bipolar	First arousal/pleasant	32	27
	First valence/unpleasant	27	32
First unipolar	First arousal/pleasant	33	28
	First valence/unpleasant	33	30

To provide a complete analysis, we assessed whether language, scale order or dimension order inuenced the ratings of participants. We found that none of these factors had more than a minor inuence on their ratings of the four assessed dimensions. An overview of all effects stemming from the differences in the rating booklets' version is provided in Table S2. The only difference between comparable booklet versions that just reached a medium effect size was that participants who found the arousal and pleasantness scales on top of each page reported a higher amount of mixed feelings, i.e. a more frequent co-occurrence of pleasant and unpleasant emotions as response to one picture. A possible explanation for this effect could be that when prompted to rate their pleasant feelings first, participants are more likely to report unpleasant ones too, whereas reporting the intensity of unpleasant feelings first seems to decrease the likelihood of also reporting pleasant feelings.

Table S2. Effect sizes for booklet language, scale order and dimension order on all rating dimensions and mixed feelings. Effect sizes with CIs not overlapping 0 are highlighted in bold face.

	d (lower 95% CI / upper 95% CI)				
Difference	Bipolar valence	Arousal	Pleasantness	Unpleasantness	Mixed feelings
English -	0.03	**0.27**	-0.13	**-0.25**	**-0.25**
German	(-0.18 / 0.25)	(0.05 / 0.48)	(-0.35 / 0.09)	(-0.47 / -0.04)	(-0.46 / -0.03)
First bipolar -	**0.23**	0.04	-0.09	-0.15	-0.07
first unipolar	(0.02 / 0.45)	(-0.17 / 0.26)	(-0.31 / 0.12)	(-0.37 / 0.06)	(-0.29 / 0.14)
First arousal/pleasant -	-0.02	0.00	-0.03	**0.36**	**0.50**
first valence/unpleasant	(-0.24 / 0.19)	(-0.22 / 0.21)	(-0.25 / 0.18)	(0.01 / 0.44)	(0.28 / 0.72)

Pre-defined picture content is reflected in pattern of mean ratings

Distinct picture categories were created to assess emotional ratings to need-of-help depictions and social context variations, including active helping. The category specific averaged responses are illustrated in Figure S1 illustrates that a-priori dened picture categories lead to distinct emotional ratings on each type of rating scale. Pictures showing birds or children within the same need of help category clearly cluster together according to both rating dimensions of both scale types. Pictures encompassing the "social context" subset rather form an independent cluster.

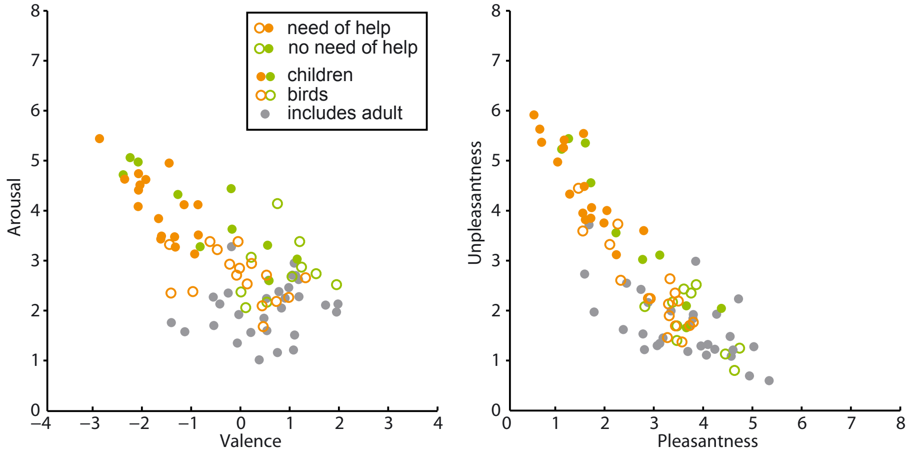

Figure S1. Means per picture on bipolar valence and arousal (A) and pleasantness and unpleasantness scales (B). Each data point corresponds to mean values of one picture. Orange dots indicate pictures showing need of help, green ones no need of help, gray ones pictures (also) showing an adult figure. Filled dots represent pictures of humans, hollow ones of birds. Note that ratings cluster according to the a-priori designed picture content categories.

Detailed results of aggregated ratings' analyses

In the main article we have shown that mean arousal and bipolar valence ratings per picture can be expressed as aggregated pleasantness and unpleasantness ratings. Specifically, arousal ratings can be inferred from the sum of pleasantness and unpleasantness ratings, bipolar valence ratings from the difference between pleasantness and unpleasantness ratings (see Figure S2).

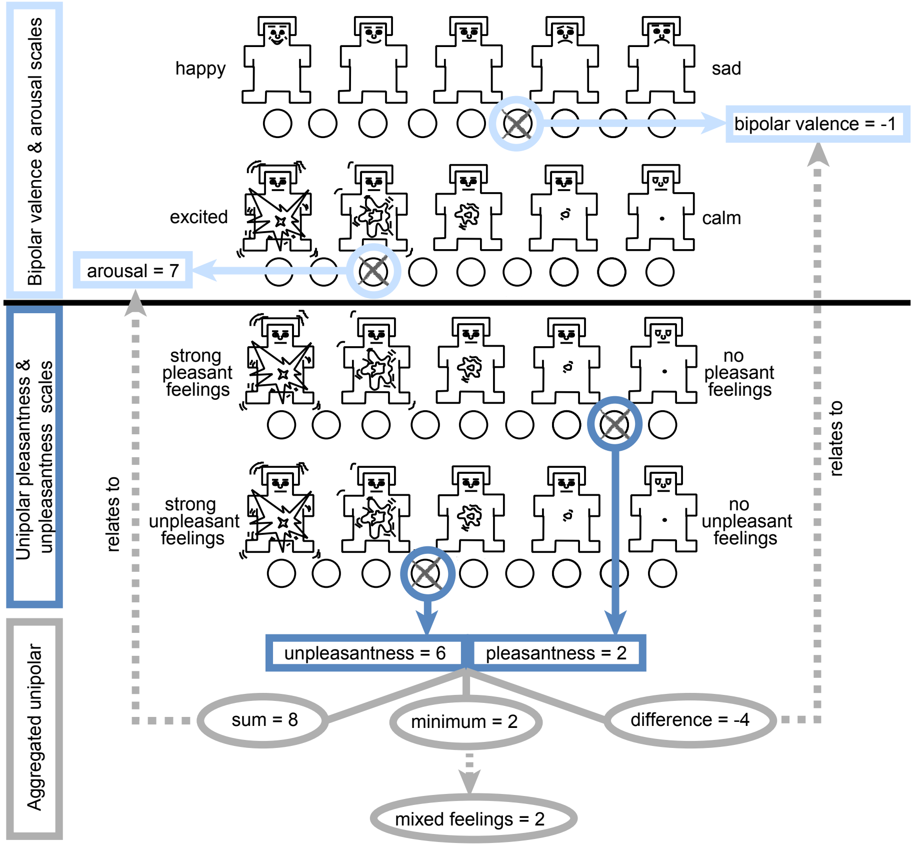

Figure S2. Example rating scales, scoring and aggregation procedures for all rating dimensions. Light blue lines refer to ratings on bipolar valence and arousal scales, dark blue ones on pleasantness and unpleasantness scales. Gray lines refer to scores obtained by combining pleasantness and unpleasantness ratings. Raw rating scores are framed with rectangles, scores derived from pleasantness and unpleasantness ratings with ellipses. Solid lines indicate direct use of raw scores, dashed ones inferences. Note that participants only used either bipolar valence and arousal scales or pleasantness and unpleasantness scales for each picture at a time.

Table S3 shows the detailed results of the analyses of aggregated pleasantness and unpleasantness ratings regarding picture content's and gender effects. Results

for the “need of help” subset are shown on top, for the “social context” subset on the bottom. Comparing the effects for the sum of pleasantness and unpleasantness ratings to the ones obtained for arousal (see Table 2 and 3 of the main article) only reveals a difference in gender effects on mean ratings across pictures. Comparing the meaningful differences emerging for unpleasantness and pleasantness ratings to the ones obtained for bipolar valence (see Table 2 and 3 of the main article), the comparison between “child alone” and “social helping” pictures is the only one that leads to another interpretation of ndings. Table 3 shows the detailed results of the analyses of aggregated pleasantness and unpleasantness ratings regarding picture content’s and gender effects. Results for the “need of help” subset are shown on top, whereas those for the “social context” subset are underneath.

Table S3. Mean values of aggregated pleasantness (P) and unpleasantness (U) ratings for the “need of help” (top) and the “social context” (bottom) stimulus subsets.

	d (lower 95% CI / upper 95% CI)	
	P − U	P + U
Need	−2.05	5.78
	(−2.61 / −1.49)	(5.53 / 6.04)
No need	0.92	5.25
	(0.50 / 1.34)	(5.02 / 5.45)
Bird	0.43	5.61
	(−0.30 / 1.16)	(5.30 / 5.91)
Child	−1.15	5.46
	(−1.69 / −0.62)	(5.25 / 5.68)
Women	−0.67	6.00
	(−1.45 / 0.10)	(5.85 / 6.15)
Men	−0.48	5.02
	(−0.97 / 0.00)	(4.76 / 5.27)
Child alone	0.55	5.43
	(−0.62 / 1.17)	(5.14 / 5.72)
Adult alone	0.80	4.41
	(−0.26 / 1.86)	(4.06/ 4.75)
Social passive	2.07	5.55
	(1.12 / 3.01)	(5.17 / 5.94)
Social helping	2.67	5.79
	(1.76 / 3.58)	(5.39 / 6.19)
Women	1.67	5.45
	(1.10 / 2.25)	(5.23 / 5.67)
Men	1.10	4.83
	(0.67 / 1.15)	(4.50 / 5.17)

Mixed feelings

The results reported so far have pointed out similarities between ratings made on the two scale types used. However, one conceptual difference between assessment of bipolar valence and unipolar pleasantness and unpleasantness is that co-occurrence of pleasant and unpleasant feelings, so called "mixed feelings", can only be measured on unipolar scales. As an indicator of so called mixed feelings the smaller value out of the pleasantness and unpleasantness ratings for each picture (min(pleasantness, unpleasantness)) was used(Schimmack, 2001; see Figure 2). Scores of mixed feelings were then averaged per picture. If the average intensity of mixed feelings is greater than 0, participants indicated to have some degree of pleasant and unpleasant feelings at the same time.

Across all pictures, the mean intensity of mixed feelings was about 1 rating step greater than 0, $M = 1.08$, [1.03, 1.14], with a narrow confidence interval indicating only little uncertainty for this finding. One explanation for this finding could be that participants avoided the extreme ends of the rating scale (Guilford, 1954). To assess this possibility, we inspected the distribution of minimal pleasantness and unpleasantness ratings and found that more than two thirds (6767 out of the 9818 ratings with valid pleasantness and unpleasantness values) were 0 or 1. Hence, there was no evidence for an aversion to use the extreme ends of the unipolar rating scales. Rather, these results suggest that participants reported genuine co-occurrence of pleasant and unpleasant emotions, i.e. mixed feelings.

The strong linear correlation between the difference of pleasantness and unpleasantness ratings and bipolar valence (see Figure 5 A of the main article) implies that bipolar valence can be regarded as a subtotal of participant's pleasant and unpleasant feelings. If this assumption is correct, mixed feelings should predominantly emerge when bipolar valence is close to zero and it can be assumed that pleasant and unpleasant feelings cancel each other out. Indeed, the intensity of mixed feelings showed a strong quadratic association with bipolar valence ratings (see Figure 3), $r(82) = -.69$, 95% CI [-.79, -.55], $R^2_{adjusted} = .47$, even though overall intensity of and variance in mixed feelings was rather low. The intensity of mixed feelings peaked around bipolar valence ratings of 0. This finding supports the interpretation that neutral bipolar valence ratings can arise because of co-occurring pleasant and un-

pleasant valence feelings. Moreover, this systematic relation reduces the likelihood that mixed feelings resulted from a bias away from the rating scales' extremes.

Taken together these results replicate previous findings (Kron et al., 2013), highlighting the existence of an emotional state, i.e. mixed feelings, which are not assessable on bipolar valence scales.

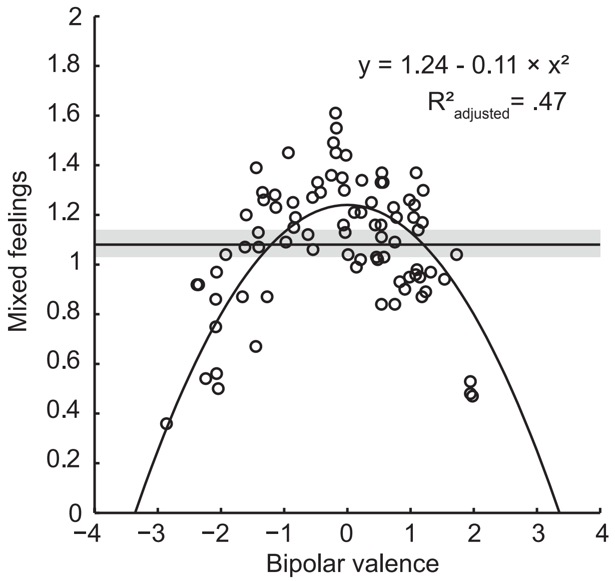

Figure S3. Relation between intensity of mixed feelings and bipolar valence. Each data point corresponds to the mean intensity of mixed feelings and mean bipolar valence ratings for one picture. The horizontal line represents the mean intensity of mixed feelings (1.08), the shaded area surrounding it the 95% CI.

Mixed feelings are scarcely affected by picture content

One last question that arises is whether mixed feelings – as an additional measure of subjective emotional experiences only assessable on unipolar scales – are influenced by picture content in the context of our study. Hence, picture content analyses were conducted for mixed feelings just as for the four explicit ratings. Results suggested that mixed feelings were affected by help-related picture content to a certain degree and mainly for ratings provided by women (see Table S4 and Figure S3).

For women only, pictures showing a child in need-of-help rather than no-need-of-help elicited somewhat fewer mixed feelings and they also tended to exhibit less mixed feelings with regard to child compared to bird pictures. Both differences were medium in size with CIs ranging also into negligible values. The difference in mixed

Table 1: Table S4. Magnitude of effects of help-related content on mixed feelings for women (left) and men (right). Effect sizes of differences with consistent direction across the entire 95% CI are highlighted in bold.

	d (lower 95% CI / upper 95% CI)	
Comparison	Women	Men
Need − no need	**−0.58**	−0.11
	(−1.14 / −0.01)	(−0.67 / 0.45)
Bird − child	**−0.58**	−0.11
	(−1.14 / −0.01)	(−0.67 / 0.45)
Need − no need (child)	**−0.95**	−0.45
	(−1.71 / −0.19)	(−1.18 / 0.28)
Need − no need (bird)	−0.03	0.32
	(−1.02 / 0.96)	(−0.68 / 1.32)
Women − men (need-of-help)	0.30	
	(−0.09 / 0.69)	
Child-alone − adult-alone	**1.23**	0.10
	(0.14 / 2.32)	(−0.89 / 1.09)
Social-passive − adult-alone	0.13	0.91
	(−0.86 / 1.12)	(−0.14 / 1.95)
Social-helping − adult-alone	−0.40	**1.11**
	(−1.40 / 0.60)	(0.04 / 2.19)
Social-passive − child-alone	−0.85	0.83
	(−1.89 / 0.19)	(−0.21 / 1.87)
Social-helping − child-alone	**−1.32**	1.02
	(−2.43 / −0.22)	(−0.04 / 2.08)
Social-passive − social-helping	0.44	−0.04
	(−0.57 / 1.44)	(−1.03 / 0.95)
Women − men (social context)	0.02	
	(−0.43 / 0.47)	

feelings for need-of-help compared to no-need-of-help depictions was evident for pictures of children not for such of birds (see Figure S4 A).

When assessing mixed feelings for pictures of the “social context” subset, women but not men reported more mixed feelings to “child-alone” compared to “adult-alone” or “social-helping” pictures (see left side of Figure S4 B). Men tended to report more mixed feelings for “social-helping” pictures than for “adult-alone” ones (see right side of Figure S4 B). Thus, social context had no consistent effects on mixed feelings and differences for both men and women displayed large condence intervals, reecting considerable uncertainty associated with the effect. Moreover, no discernible social context category stood out for either gender, however it seems that gender differences with regard to the presence and the intensity of mixed feelings

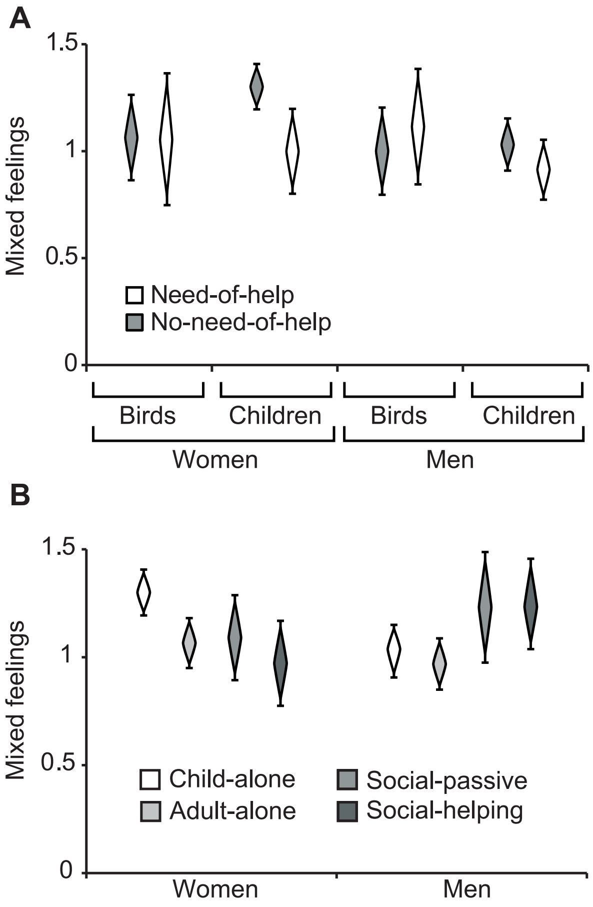

Figure S4. Intensity of mixed feelings in the “need-of-help” (A) and “social context” subset (B). CIs of mixed feelings’ intensities are shown separately for women (left side of each graph) and men (right side of each graph). Effects of need-of-help content are shown in the top panel, with white cat’s eyes representing means for need-of-help pictures and gray ones representing means for no-need-of-help pictures. Effects of social contextual content are shown in the bottom panel with white cat’s eyes representing “child-alone” pictures, light gray ones “adult-alone” pictures, middle gray ones “social-passive” and dark gray ones for “social-helping” pictures. The length of cat’s eyes indicates 95% confidence intervals.

to certain content categories might be easier to uncover using unipolar pleasantness and unpleasant scales rather than valence ratings.
