# Supplementary material for: Does it matter how you ask? Self-reported emotions to depictions of need-of-help and social context
Source: BMC Psychol. 2015 Apr 7;3(1):10. doi: 10.1186/s40359-015-0066-3 (PMC4403975; doi:10.1186/s40359-015-0066-3)
Supplement: Additional file 3 — Example rating booklet exert. Exemplary instruction and first rating pages for one of the English rating booklets versions. [file 40359_2015_66_MOESM3_ESM.pdf]

Participant ID: ______

Consent form
Participation in a perception study
assessing characteristics of a recently designed stimulus set

In this perception study lasting approximately 60 minutes you will see comic pictures of humans and birds on a projection screen. Humans and birds will be shown in an everyday situation (e.g. reaching for an object) which they can either master on their own or in which they need help. It will be your task to indicate on a rating scale how you felt while looking at these pictures. At the beginning of the study, we will also ask you to indicate the subjectively perceived gender of the adults shown on N different pictures.

Your participation helps us to investigate the emotional and perceptual attributes of our stimulus set as perceived by adults. What is more we would like to find out whether responses differ between groups of people with varying characteristics. Using these insights we will be able to design new experiments assessing the development of empathy and the willingness to help and we will be able to further develop our stimulus set.

Your answers will be treated strictly anonymously regarding recording, storage, as well as further processing. In addition to your ratings of the pictures presented we will also collect some demographic information. The questionnaire concerning these questions are included in your booklet, you will have time to answer them at the end of the study. All answers are voluntary as is the participation in this study.

Participation in this study does not pose any physical or psychological risks to you. You are free to terminate the experiment at any time without causing any disadvantages for you. At the end you will receive a small present in appreciation.

I have been fully informed about aims, procedure, duration and potential risks of this study and had ample opportunity to ask questions.
I approve taking part in the study described above (taking approx. 1h including instructions). I do not disagree with the anonymous storage of the data collected.

Kleve, _______________, ...
(date) (signature)

☐I would like to be informed about the study's results:

HOCHSCHULE RHEIN-WAAL
Rhine-Waal University of Applied Sciences

NeoHelp

Evaluation

Participant ID: 201

We appreciate your participation in this experiment. For about the next 10 minutes, you will be looking at different pictures projected on the screen in front of you. Each picture will depict an adult, sometimes along with a child. Please decide for each picture whether the ***adult*** depicted is in your opinion a man or a woman. There are no right or wrong answers, so simply respond as honestly as you can.On this sheet, you can see that there will be three options to choose from: “Man”, “Woman” and “I don't know”. Please select one of these three options for each picture and do not omit any picture because you are unsure about your opinion. Simply chose the option that applies best for you.

So if you think the adult depicted shows a Man, mark the circle below the "Man" symbol with an "X" like here:

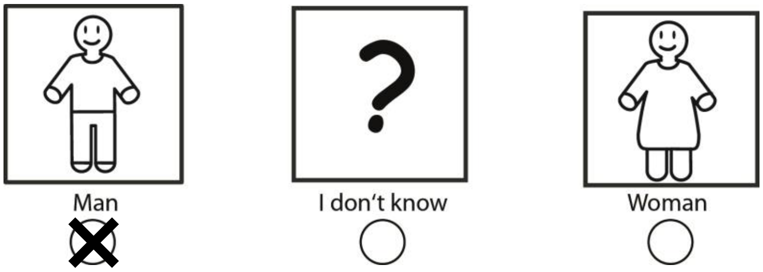

If in your opinion the adult depicted was a woman, place an "X" below the "Woman" symbol, like here:

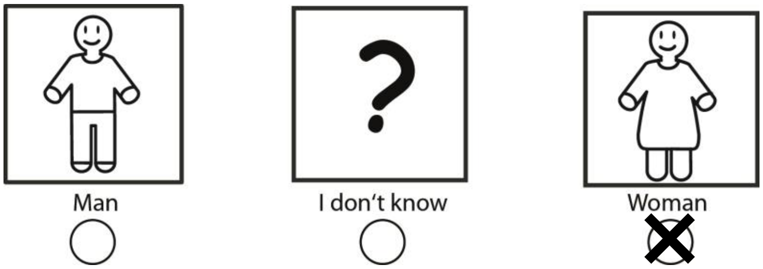

If you have truly no idea whether the adult depicted was a man or a woman, place an "X" below the question mark symbol in the middle, like below.

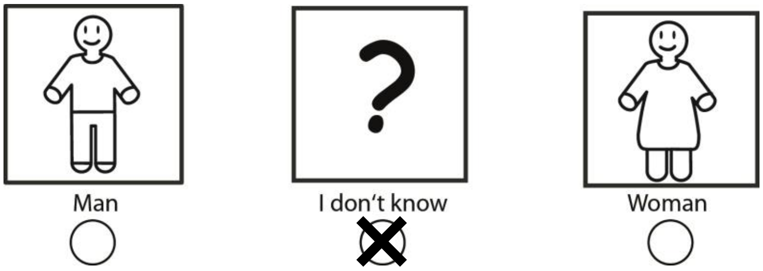

For some of the pictures the adult's gender might immediately be evident to you, for other pictures the gender of the adult depicted may be very ambiguous.Your rating of each picture should reflect your immediate personal experience, and no more. Please rate each one **as you perceived the gender while watching the picture**. Do not leave the scale blank or place an "X" anywhere else but inside the three circles.

The procedure will be as follows: Before each of the pictures which you will rate, there will be a warning slide that indicates the letter of the upcoming picture. At these times, you should always be certain that the picture letter corresponds to the ratings letter. For example, when you see "Rate the next picture F", you should turn to the scale with “picture F” written on its left side. The warning slide should also prompt you to quickly complete the previous rating and pay close attention to the screen.

It is important that your eyes are directed towards the screen when the pictures to be rated are shown. You'll have only a few seconds to watch each picture. Please look at the picture for the entire time it is on. Make your ratings immediately after the picture is removed. If, for some reason, you should miss viewing any picture, please leave that rating scales blank. Remember: The number besides your ratings must always have the same letter as the picture.

After each picture, you'll see projected “Please rate the adult's gender”. Take this time to record your experience of the adult's gender on your sheet. It is very important not to dwell on your ratings of the pictures, since there will not be much time. Also remember that you will need to check the correct letter given on the warning slide for the next trial.

We are interested in your own personal ratings of the depicted adults' gender. Therefore, please don't make any comments which might influence the ratings that other people make. You can understand how this might bias our results.

Just a reminder before we begin: when the warning slide comes on, make sure the picture letter on the screen and besides your rating scale match. Then view the picture slide for the entire time it is on. After the picture is off, make your rating as quickly as possible and get ready for the next picture. It is important that we have information from each of you on all of these pictures. There are no right or wrong answers; so rate every picture according to your impression while viewing it. The experiment will start immediately after the last question concerning these instructions has been answered.

Picture A

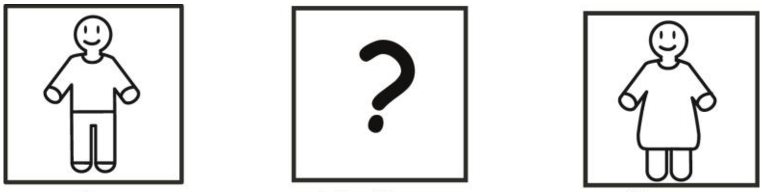

Man I don't know Woman

PictureB

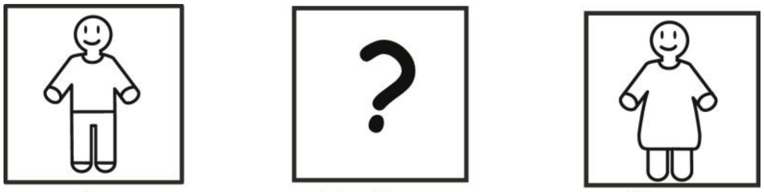

Man I don't know Woman

PictureC

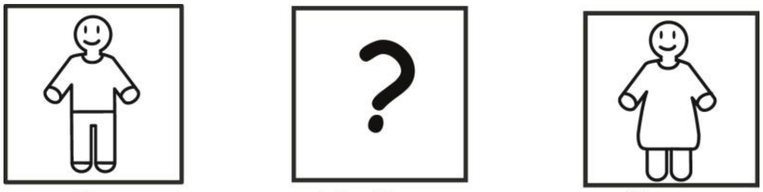

Man I don't know Woman

PictureD

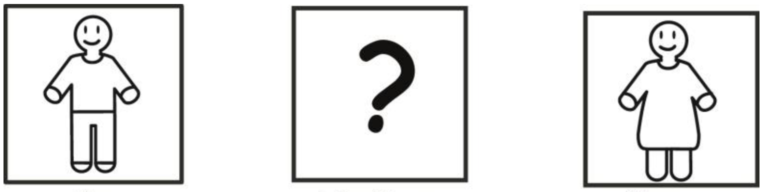

Man I don't know Woman

PictureAC

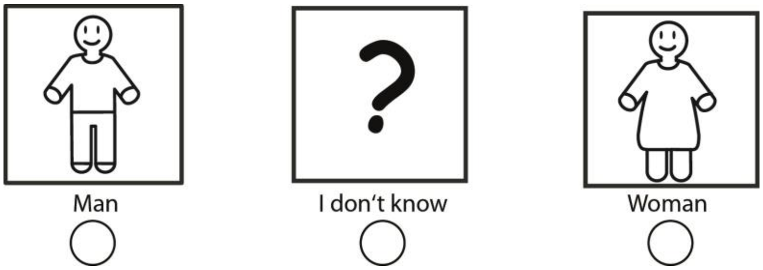

PictureAD

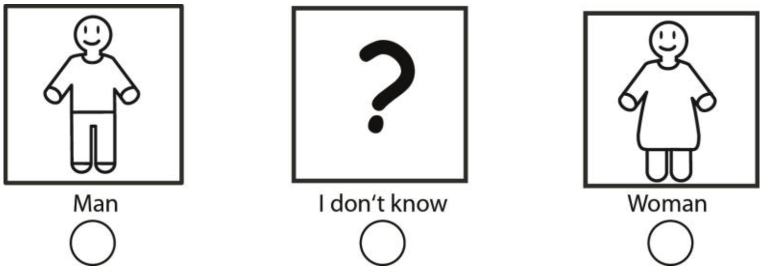

Thank you!

This was the first part of the experiment.

Please read the following instructions.

For about the next 30 minutes, you will be looking at different pictures projected on the screen in front of you, and you will be rating each picture in terms of **how it made you feel while viewing it.** There are no right or wrong answers, so simply respond as honestly as you can.

On this sheet, you can see sets of 5 figures, each arranged along a continuum. We call this set of figures SAM, and you will be using these figures to rate how you felt while viewing each picture. You will use ratings for each picture that you observe. SAM shows two different kinds of feelings: Happy vs. Unhappy and Excited vs. Calm. Each SAM figure varies along each scale.

The first SAM scale below is the happy - unhappy scale, which ranges from a smile to a frown. At one extreme of the happy vs. unhappy scale, you felt happy, pleased, satisfied, contented, hopeful. If you felt completely happy while viewing the picture, you can indicate this by placing an "X" below the figure at the left, like this:

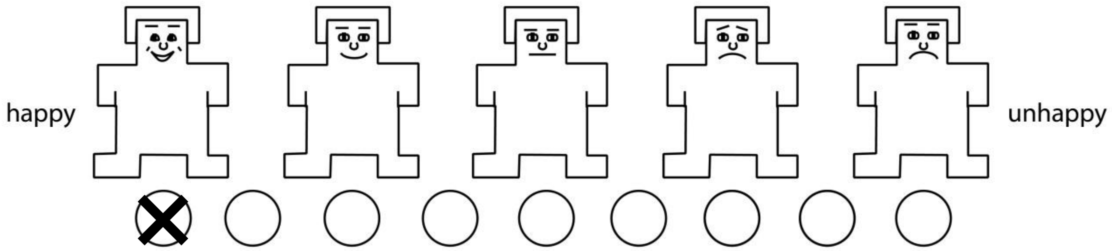

The other end of the scale is when you felt completely unhappy, annoyed, unsatisfied, melancholic, despaired, bored. You can indicate feeling completely unhappy by placing an "X" below the figure at the right, like this:

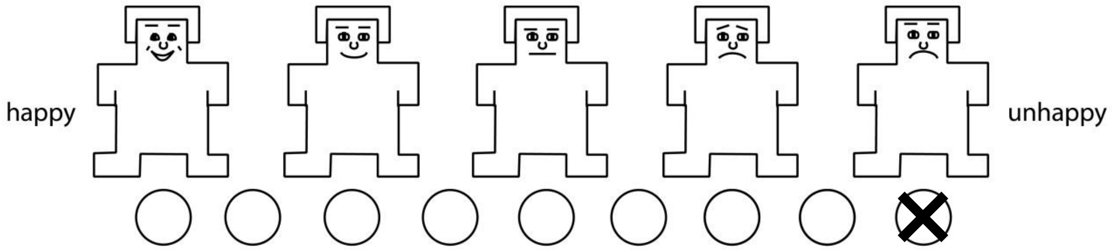

The figures also allow you to describe intermediate feelings of pleasure, by placing an "X” below any of the other pictures. If you felt completely neutral - neither happy nor sad, place an "X" below the figure in the middle.

If, in your judgment, your feeling of pleasure or displeasure falls between two of the pictures, then place an "X" between the figures, like below. This permits you to make more finely graded ratings of how you feel in reaction to the pictures.

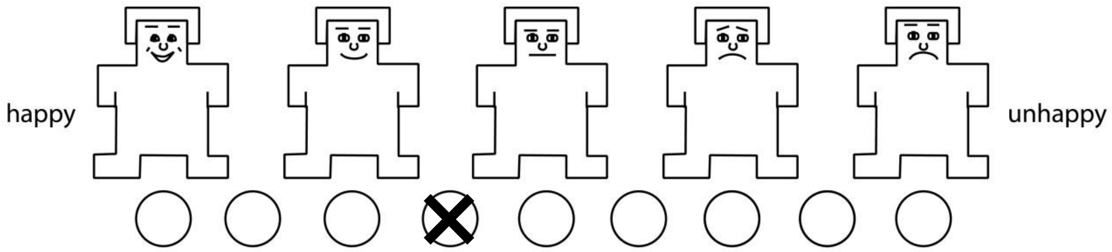

The excited vs. calm dimension is the second type of feeling displayed here. At one extreme of the scale you felt stimulated, excited, frenzied, jittery, wide-awake, aroused. If you felt completely aroused while viewing the picture, place an "X" below the figure at the left of the row, like this:

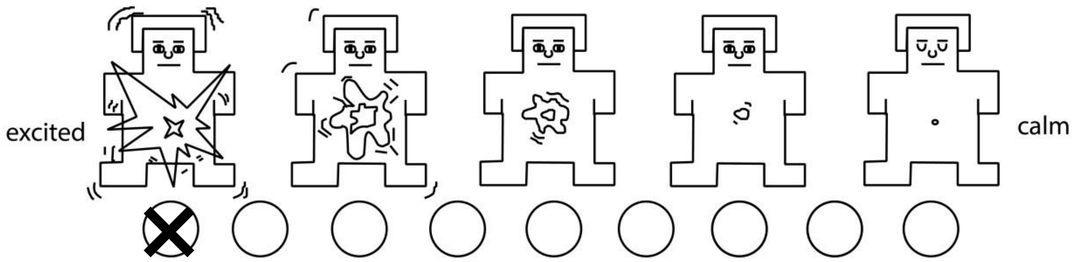

On the other hand, at the other end of the scale, you felt completely relaxed, calm, sluggish, dull, sleepy, unaroused. You can indicate you felt completely calm by placing an "X" below the figure at the right of the row, like this:

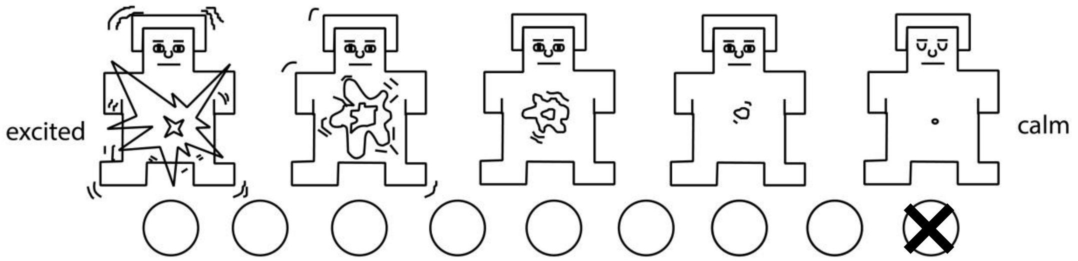

As with the happy-unhappy scale, you can represent intermediate levels by placing an "X" below any of the other figures. If you are not excited at all nor calm at all, place an "X" below the figure in the middle of the row.

Again, if you wish to make a more finely tuned rating of how excited or calm you feel, place an "X" between the pictures, like this:

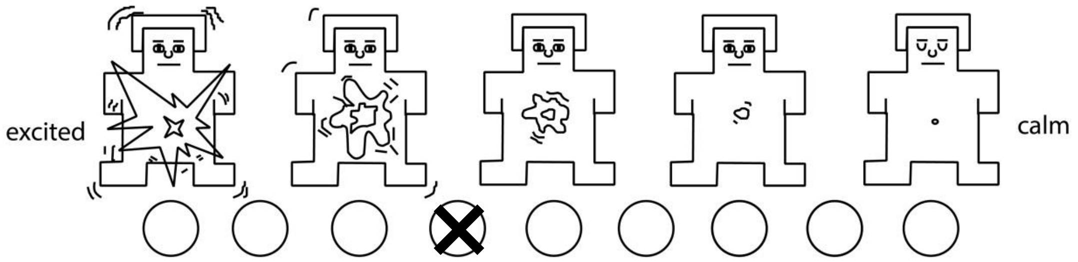

Some of the pictures may prompt emotional experiences; others may seem relatively neutral. Your rating of each picture should reflect your immediate personal experience, and no more. Please rate each one AS YOU ACTUALLY FELT WHILE YOU WATCHED THE PICTURE.

The procedure will be as follows: Before each of the pictures which you will rate, there will be a warning slide that indicates the number of the upcoming picture. At these times, you should always be certain that the picture number corresponds to the ratings number. For example, when you see "Rate the next picture #10", you should turn to those SAM scales with the heading “picture #10”. The warning slide should also prompt you to quickly complete the previous rating and pay close attention to the screen.

It is important that your eyes be directed towards the screen when the pictures to be rated are shown. You'll have only a few seconds to watch each picture. Please look at the picture for the entire time it is on and make your ratings immediately after the picture is removed. If, for some reason, you should miss viewing any picture, please leave that rating scales blank. Remember: The number above your ratings must always have the same number as the picture.

After each picture, you'll see projected “Please rate the picture on both dimensions”. Take this time to record your emotional experience of the picture on your sheet. It is very important not to dwell on your ratings of the pictures, since there will be not much time. Also remember that you will need to check the correct number given on the warning slide for the next trial.

Please note that the 2 dimensions are not presented in the same order on each page. Look at the page after these instructions to see that the dimensions are presented in different orders.

We are interested in your own personal ratings of the pictures. Therefore, please don't make any comments which might influence the ratings that other people make. You can understand how this might bias our results.

Before the actual experiment begins, there will be examples of the kinds of pictures you will be viewing and rating. The page after these instructions is a sample rating sheet on which you can practice rating the following pictures, all on the same sheet. This is just to help you get a feel for how the ratings are done.

Just a reminder before we begin: when the warning slide comes on, make sure the picture number on the screen and above your SAM rating scales match. Then view the picture slide for the entire time it is on. After the picture is off, make your ratings on both dimensions as quickly as possible and get ready for the next picture. It is important that we have information from each of you on all of these pictures. There are no right or wrong answers; so rate every picture on both dimensions.

Example picture #1

excited vs. calm:

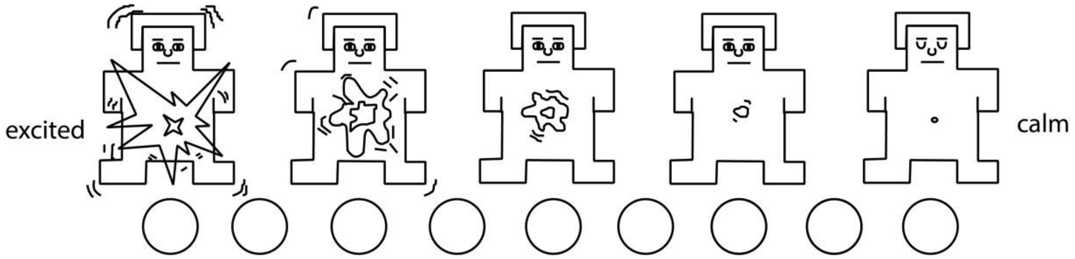

happy vs. unhappy:

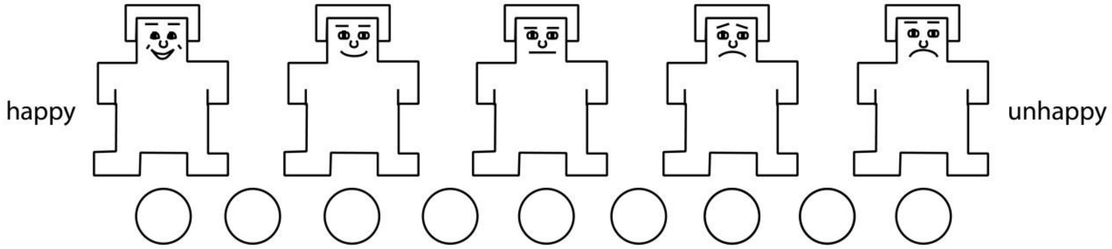

Example picture #2

happy vs. unhappy:

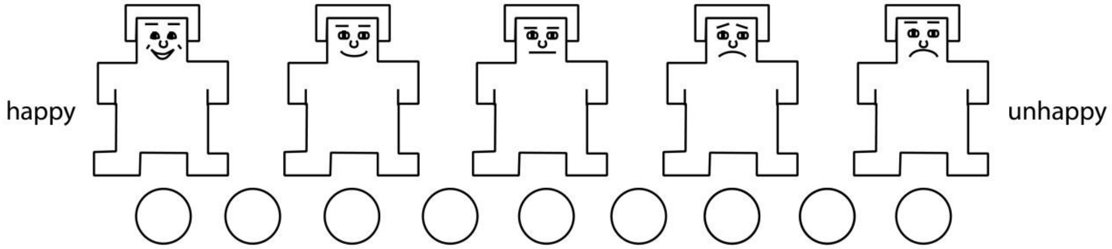

excited vs. calm:

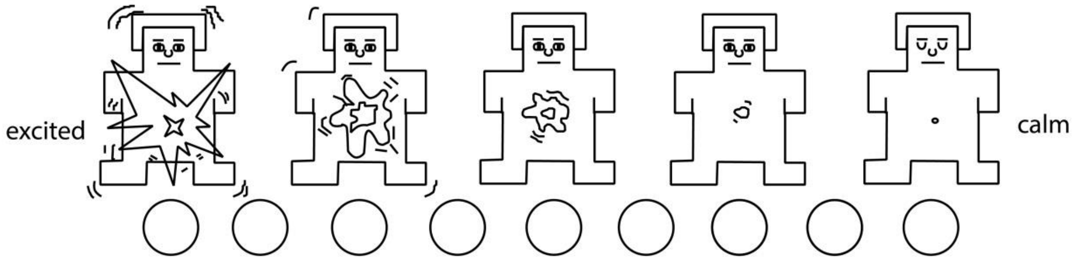

Picture #1

excited vs. calm:

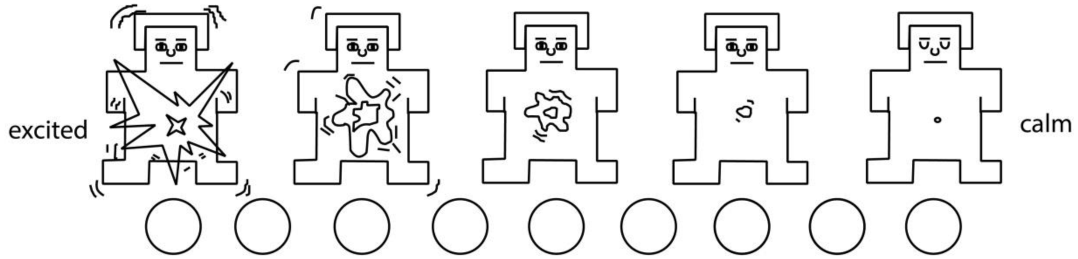

happy vs. unhappy:

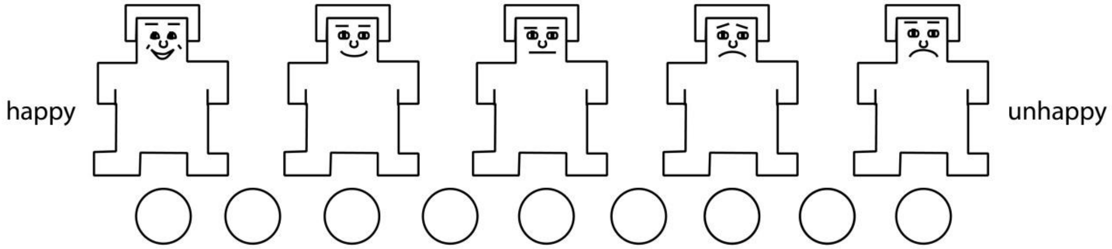

Picture #2

happy vs. unhappy:

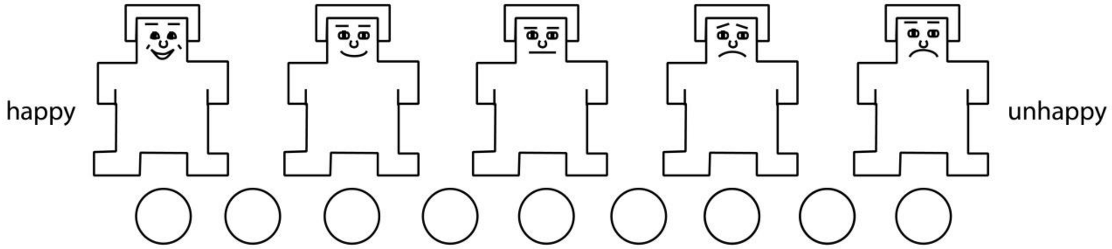

excited vs. calm:

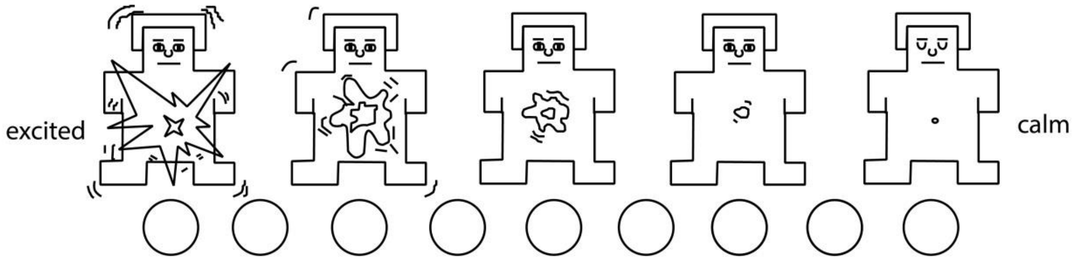

You have now finished rating half of the pictures using the SAM scales displaying happy vs. unhappy and excited vs. calm feelings.

For the next half of the picture you will use ***different SAM scales***, so please read the following instructions carefully. The SAMs you will use now show ***the intensity of a given feeling from strong to none***. Each SAM figure varies along each scale.

The first SAM scale below is the pleasant feelings scale. At one extreme of the strong pleasant feelings vs. no pleasant feelings scale, you felt strongly happy, pleased, satisfied, contented, hopeful. If you felt strong pleasant feelings while viewing the picture, you can indicate this by placing an "X" below the figure at the left, like this:

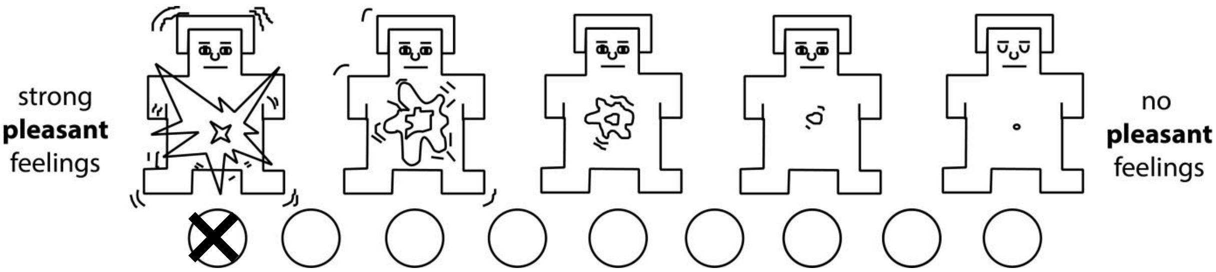

The other end of the scale is when you had ***no*** happy, pleased, satisfied, contented, hopeful feelings. You can indicate that you did not have any pleasant feelings by placing an "X" on the figure at the right, like this:

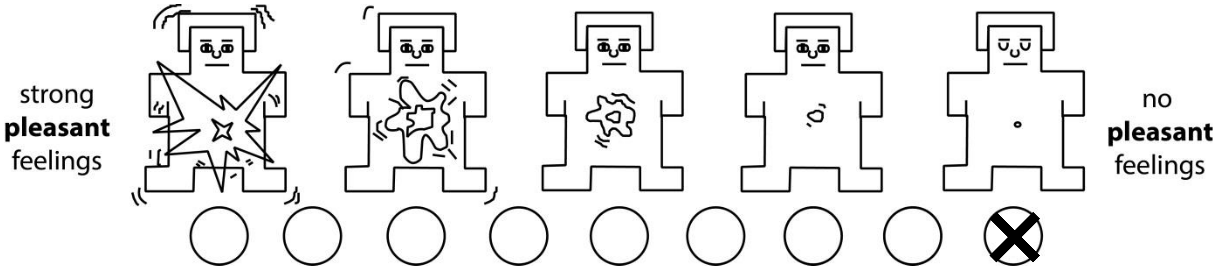

The figures also allow you to describe intermediate levels of pleasant feelings, by placing an "X" below any of the other pictures. If you felt completely neutral, neither strongly happy nor not happy at all, place an "X" below the figure in the middle.

If, in your judgment, your feeling of pleasure falls between two of the pictures, then place an "X" between the figures, like below This permits you to make more finely graded ratings of how you feel in reaction to the pictures.

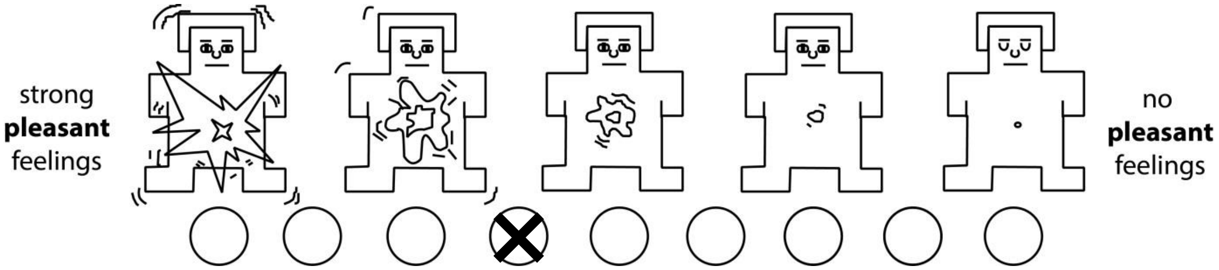

The strong unpleasant feelings vs. no unpleasant feelings scale is the second type of feeling displayed here. At one extreme of the scale you felt completely, unhappy, annoyed, unsatisfied, melancholic, despaired, bored. You can indicate having had strong unpleasant feelings by placing an "X" on the figure at the left of the row, like this:

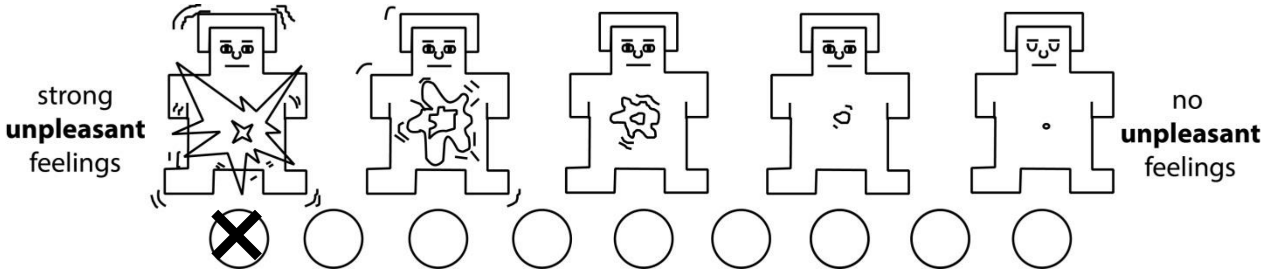

On the other hand, at the other end of the scale, you felt ***not*** unhappy, annoyed, unsatisfied, melancholic, despaired, bored. You can indicate you felt ***no*** unpleasant feelings by placing an "X" below the figure at the right of the row, like this:

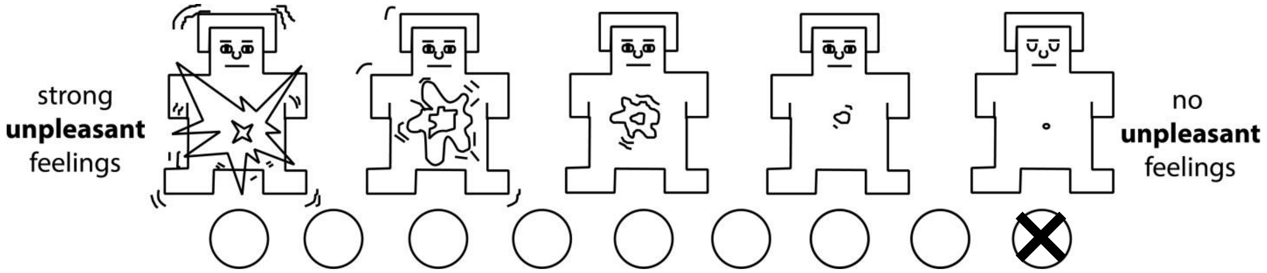

As with the strong pleasant feelings vs. no pleasant feelings scale, you can represent intermediate levels by placing an "X" below any of the other figures. If you felt completely neutral, neither strongly unhappy nor not unhappy at all, place an "X" below the figure in the middle of the row.

Again, if you wish to make a more finely tuned rating of how strong your unpleasant feelings were, place an "X" between the pictures, like this:

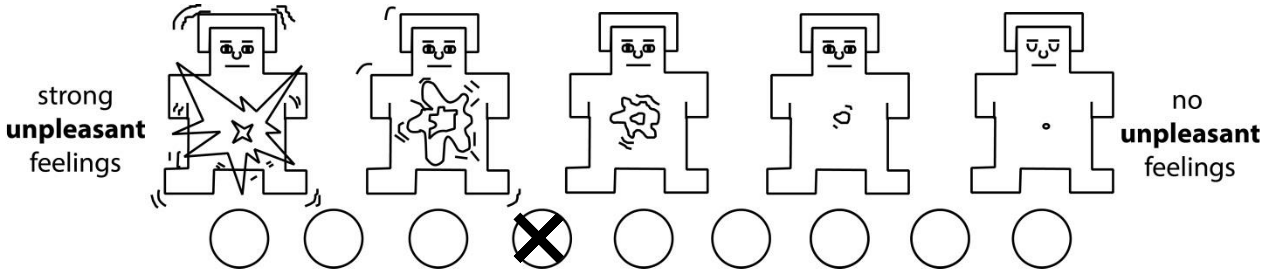

The procedure will be the same as for the first half of the experiment.

Just a reminder before we begin: Rate each picture according to how strong your pleasant and how strong your unpleasant feelings were while viewing the picture. There are no right or wrong answers; so please rate every picture on both dimensions.

Picture #43

pleasant feelings:

strong **pleasant** feelings

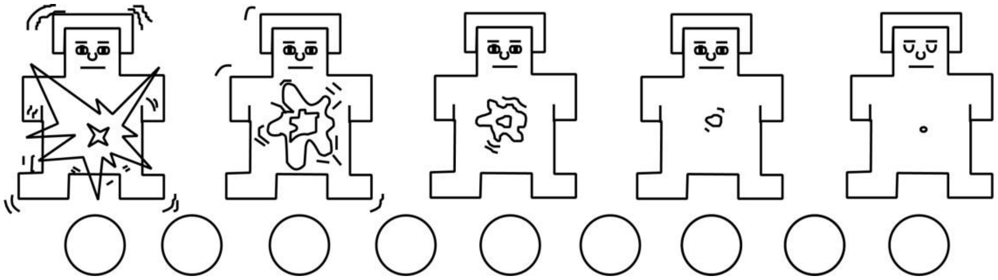

no **pleasant** feelings

unpleasant feelings:

strong **unpleasant** feelings

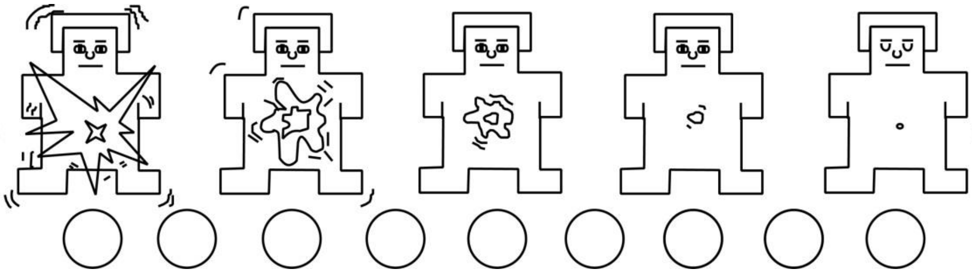

no **unpleasant** feelings

Picture #44

unpleasant feelings:

strong **unpleasant** feelings

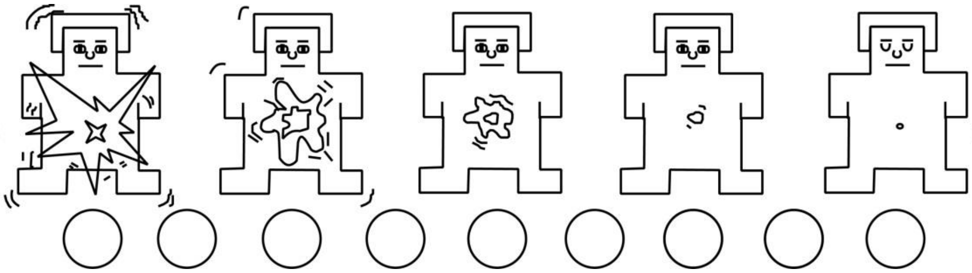

no **unpleasant** feelings

pleasant feelings:

strong **pleasant** feelings

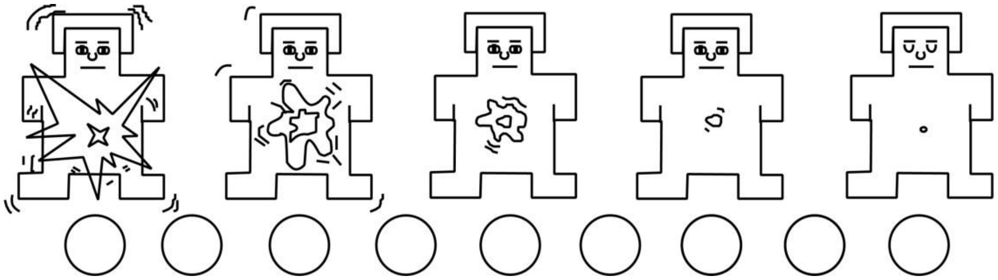

no **pleasant** feelings

Picture #83

pleasant feelings:

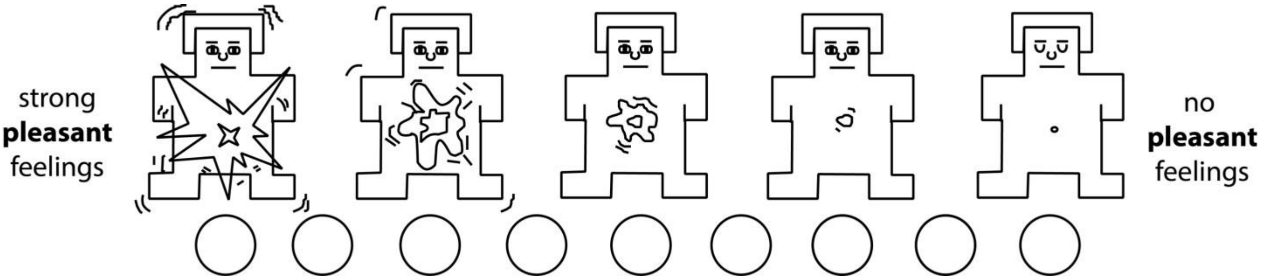

unpleasant feelings:

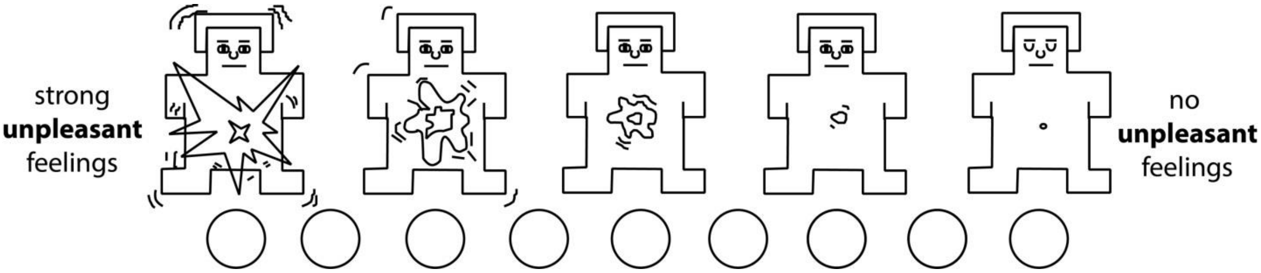

Picture #84

unpleasant feelings:

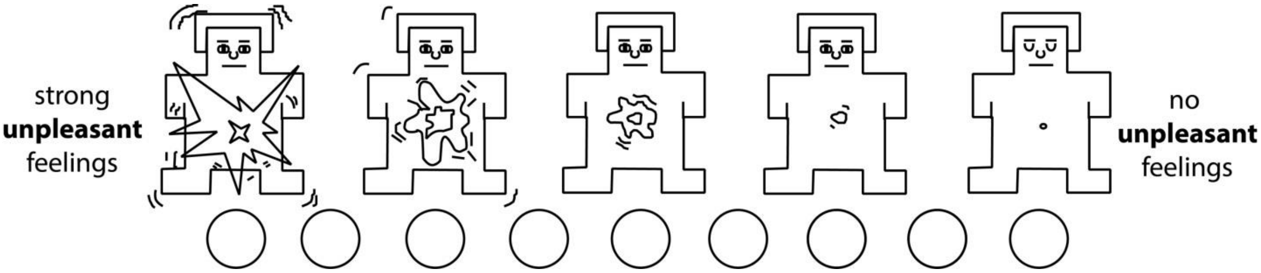

pleasant feelings:

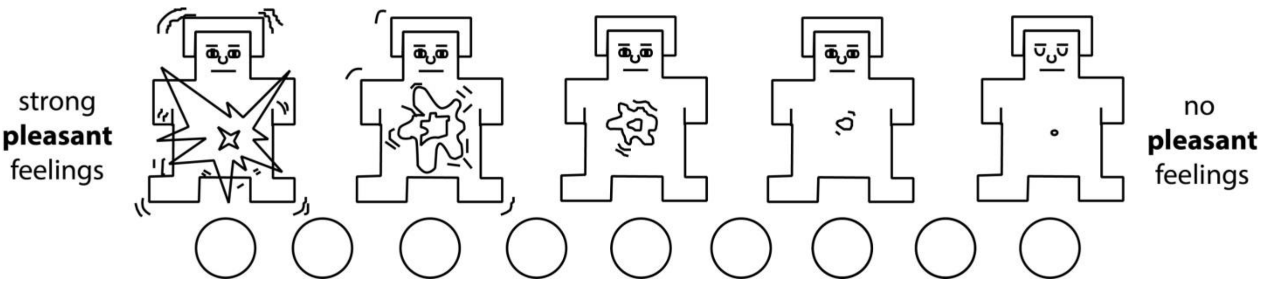

Thank you for your participation. You have now seen all pictures and have rated them. Finally we would like to know whether you noticed anything special during the experiment. Your answers will help us finding out whether we should change the experiment in the future and whether the content of the pictures used is clear enough.

Did you rate each picture honestly regarding your own opinion/feelings? ☐yes ☐no

If no, which one(s)? ____________________

To what else did your rating refer to? ____________________

____

Was there any picture that you considered strange? ☐yes ☐no

If yes, which one(s)? ____________________

Why? ____________________

Did you ever have the feeling that you have to give a certain "correct" answer other than your own opinion/feelings? ☐yes ☐no

If yes, for which one(s)?____________________

Why? ____________________

Did you ever think a picture was completely unrealistic? ☐ yes ☐ no

If yes, which one(s)? ____________________

What was unrealtistic? ____________________

Short demographic questionnaire

This short questionnaire will aid us determining which characteristics and environmental factors might influence the ratings of our stimulus set.

All answers are voluntary and will be stored and processed strictly anonymously.

- age: _______ date of birth: ______ ._____ . __________
- gender: ☐male ☐ female ☐other
- handedness (predominantly): ☐ right ☐left ☐ both
- field of studies: ☐ Frühkindliche Bildung Semester:__________________

  ☐Alternativer Tourismus Semester: __________________

  ☐ International Business & Social Sciences Semester: ______

  ☐ International Relations Semester:__________________

  ☐ anderer: _____________________________________ Semester:_____
- citizenship: own:____

  father:_________________________ mother:____________________
- country of birth: ____
- native language: ☐English only

  ☐English and one/more other languages, (which one(s)?)_______________

  ☐one native language other than English (which one?) _____________

  ☐more than one native language other than English (which ones):

  ____
- visual acuity: ☐normal

  ☐corrected to normal (contacts/glasses):

  ☐wearing them at the moment ☐not wearing them

  ☐uncorrected:

  left: __________ dpt, right: ____________ dpt

Thank you!
